# Zinc finger nuclease-based double-strand breaks attenuate malaria parasites and reveal rare microhomology-mediated end joining

**DOI:** 10.1186/s13059-015-0811-1

**Published:** 2015-11-17

**Authors:** Mirko Singer, Jennifer Marshall, Kirsten Heiss, Gunnar R. Mair, Dirk Grimm, Ann-Kristin Mueller, Friedrich Frischknecht

**Affiliations:** Integrative Parasitology, Center for Infectious Diseases, University of Heidelberg Medical School, Im Neuenheimer Feld 324, 69120 Heidelberg, Germany; Parasitology, Center for Infectious Diseases, University of Heidelberg Medical School, Im Neuenheimer Feld 324, 69120 Heidelberg, Germany; MalVa GmbH, Heidelberg, Germany; Virology, Center for Infectious Diseases, University of Heidelberg Medical School, Im Neuenheimer Feld 267, 69120 Heidelberg, Germany; German Center for Infectious Diseases, Im Neuenheimer Feld 324, 69120 Heidelberg, Germany

**Keywords:** Apicomplexa, *Plasmodium*, Malaria, Genetically attenuated parasites, Vaccine, Zinc-finger nucleases, Alternative end joining, Microhomology-mediated end joining, Alternative DNA repair, DSB

## Abstract

**Background:**

Genome editing of malaria parasites is key to the generation of live attenuated parasites used in experimental vaccination approaches. DNA repair in *Plasmodium* generally occurs only through homologous recombination. This has been used to generate transgenic parasites that lack one to three genes, leading to developmental arrest in the liver and allowing the host to launch a protective immune response. While effective in principle, this approach is not safe for use in humans as single surviving parasites can still cause disease. Here we use zinc-finger nucleases to generate attenuated parasite lines lacking an entire chromosome arm, by a timed induction of a double-strand break. Rare surviving parasites also allow the investigation of unconventional DNA repair mechanisms in a rodent malaria parasite.

**Results:**

A single, zinc-finger nuclease-induced DNA double-strand break results in the generation of attenuated parasite lines that show varying degrees of developmental arrest, protection efficacy in an immunisation regime and safety, depending on the timing of zinc-finger nuclease expression within the life cycle. We also identify DNA repair by microhomology-mediated end joining with as little as four base pairs, resulting in surviving parasites and thus breakthrough infections.

**Conclusions:**

Malaria parasites can repair DNA double-strand breaks with surprisingly small mini-homology domains located across the break point. Timely expression of zinc-finger nucleases could be used to generate a new generation of attenuated parasite lines lacking hundreds of genes.

**Electronic supplementary material:**

The online version of this article (doi:10.1186/s13059-015-0811-1) contains supplementary material, which is available to authorized users.

## Background

Malaria poses a heavy health and economical burden in endemic countries and is caused by apicomplexan parasites of the genus *Plasmodium*. More than one billion people are at risk of infection and fatalities occur mainly in children aged under 5 years [1]. *Plasmodium* infection results in a weak and mostly short-lived semi-immunity resulting in frequent re-infection with slowly decreasing severity under continuous exposure [2, 3]. In the field, *Plasmodium falciparum* parasites have readily acquired resistance to most antimalarial drugs by both point mutations and gene copy number variations [4, 5]. In vitro drug resistance selection was shown to be initiated by duplications of large genomic regions flanked by naturally occurring A/T repeats. After the initial duplication, this locus was further expanded by more efficient homology-based mechanisms [6]. This massive expansion confers some drug resistance but also has a high metabolic cost for the parasite, as multiple unrelated genes are co-amplified. It is believed that the process of copy number variation, followed by point mutations conferring resistance and a subsequent homology-based de-amplification, leads to the fast appearance of drug-resistant parasites in the field [7].

Malaria parasites are transmitted during a bite of infected female *Anopheles* mosquitoes. Injected into the skin these *Plasmodium* sporozoites rapidly migrate and enter blood vessels [8]. They disperse within the blood through the mammalian body and specifically arrest in the liver, where they invade hepatocytes, replicate and differentiate into red blood cell-invading forms that ultimately cause the disease [9]. No infection-blocking vaccine is currently available for malaria, as is the case for all other human diseases caused by eukaryotic pathogens [10]. The gold standard of experimental immunisation against *Plasmodium* and other parasites like *Schistosoma* is repeated infection with radiation-attenuated parasites [11, 12]. In the case of *Plasmodium*, γ-irradiation of sporozoites is assumed to cause random double-strand breaks (DSBs) in the genome as well as RNA damage, resulting in surviving parasites which still actively invade liver cells but soon arrest in development and elicit a protective immune response [13].

Attempts have been made to replicate this phenotype in a consistent and genetically defined manner by generating deletion mutants of genes essential for liver stage development [14–16]. These genetically attenuated parasites (GAPs) are generally more consistent in their timing of arrest than radiation attenuated sporozoites and are a reproducible, standardised source for attenuated parasites. GAPs have been classified into early and late liver stage-arresting parasites [17–19]. Multiple genes have been exploited for the generation of GAPs in rodent malaria parasites [20]. Triple immunisation with live GAP sporozoites can elicit sterile protection against a subsequent challenge with wild-type (WT) parasites [14]. However, many GAPs show breakthrough infections during immunisation, resulting in a full-blown pathological blood-stage infection, and these have not always been consistent in the two main model parasites, *Plasmodium berghei* and *Plasmodium yoelii* [16]. Transfer of data obtained in rodent-infecting *Plasmodium* models to the major human malaria parasite *P. falciparum* has, therefore, been challenging. It is currently assumed that multiple (up to triple) gene deletions in a single parasite are needed to reduce breakthrough infections to zero. However, a final verdict is still open due to the limitation of the testable range for parasites and mice/human volunteers under pre-clinical conditions [21, 22].

**Fig. 1:**
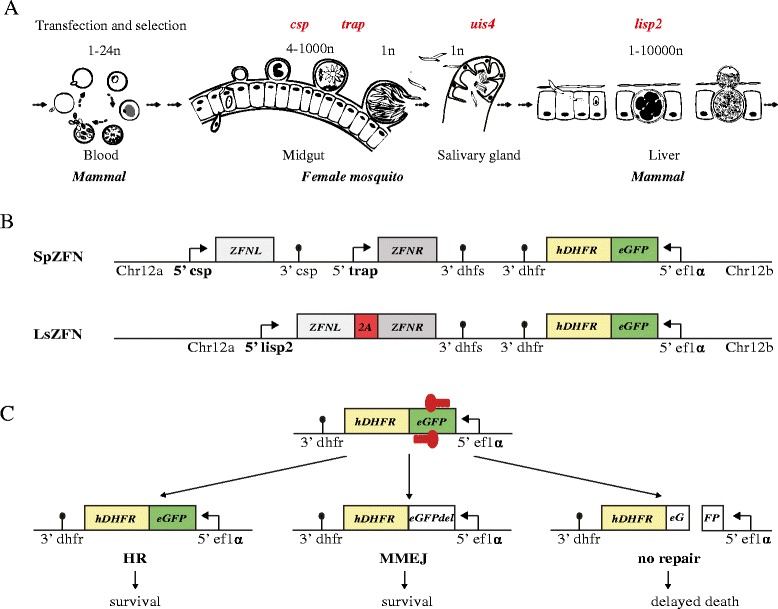
Zinc-finger nuclease (ZFN)-mediated double-strand breaks (DSBs) and potential repair in the context of the *Plasmodium* life cycle. **a** Parts of the life cycle relevant for this study with genome copy number of various stages indicated. Gene names of the used promoters are depicted in *red* at the point of their respective activation. **b** Design of SpZFN and LsZFN transgenic parasite lines. *Chr12a* and *Chr12b* are the sequences used for homologous integration into the genome. The selection marker hDHFR (human dihydrofolate reductase; *yellow*) is expressed as a fusion protein with eGFP (enhanced green fluorescent protein; *green*) under the constitutively active ef1α promoter. The target sequence of the ZFNs is present within the *egfp* gene. ZFNL and ZFNR are expressed under the control of the two promoters of *csp* and *trap* in SpZFN and the promoter of *lisp2* in LsZFN. Both *zfn* genes are fused with a 2A skip peptide (*red*) in LsZFN. **c** ZFN-induced DSB and possible post-DSB outcomes are depicted. Homologous recombination (*HR*) can restore the original locus only if the genome copy number is >1 and restores ZFN binding sites, since no sufficient homology regions are flanking the break site. Potential microhomology-mediated end joining (*MMEJ*) can repair the DSB, resulting in small gene deletions that can be detected by loss of fluorescence. Parasites that fail to repair the DSB are expected to die

The genome of *Plasmodium* is haploid, with the exception of the zygote (2n1c) and the resulting ookinete (2n2c), which is the form that invades the mosquito midgut (Fig. 1a) and transforms into the diploid oocyst. Replicating stages of *Plasmodium*, the oocyst during sporogony as well as the liver- and intra-erythrocytic stages contain nuclei with multiple (sometimes thousands) genome copies in a single cell. Genetic modification in *Plasmodium* utilises transfection of plasmid DNA and its exclusive integration via homologous recombination (HR) in blood stage parasites [23, 24]. In contrast, the related apicomplexan parasite *Toxoplasma gondii* mainly employs non-homologous end joining (NHEJ) as the primary DNA repair pathway. In this species genes involved in the NHEJ pathway, including Ku70/80, were readily identified. A *T. gondii* parasite line lacking Ku80 can only perform HR, thus allowing efficient targeted gene modification via HR [25]. Genes involved in NHEJ have so far not been identified in any *Plasmodium* species [26, 27]. Also, recent data suggest that alternative DNA repair can occur in *P. falciparum* [27].

The generation of live attenuated sporozoites suffers from the lack of a clear list of target genes that, when deleted, can be predicted to cause the desired effect, i.e. developmental arrest in the liver without breakthrough infections. We propose that a well-timed and tightly regulated expression of zinc-finger nucleases (ZFNs) will result in a DSB that the parasites — in the absence of the NHEJ machinery or a plasmid repair template for HR — cannot repair [28]. This should result in parasite death after the next nuclear division, as they would lose the part of the cut chromosome lacking the centromere and thus tens to hundreds of genes. Properly engineered and timed, such parasites could be used as next-generation attenuated experimental vaccines. In addition, the set of parasites needed to evaluate attenuation capacity should allow us to probe alternative DNA repair mechanisms.

## Results

### Generation of *P. berghei* parasites expressing ZFNs in mosquito and liver stages

Many genes in *Plasmodium* are expressed only during a certain developmental stage of the parasite [29], providing proteins needed for specific functions such as adhesion to a host cell. In order to induce ZFN-mediated DSBs during mosquito-to-mouse transmission, we selected four well-characterised, stage-specific promoters to control ZFN expression. These promoters provide defined and tightly regulated transcription profiles in the mosquito and during the liver stage but are otherwise silent. The promoter of the gene encoding the circumsporozoite protein (CSP) is active from the mid-oocyst stage to the liver stage [30] (and our own unpublished data) (Fig. 1a). The promoter of *trap*, encoding the thrombospondin-related anonymous protein (TRAP), is active from the late oocyst stage to the mature sporozoites, but not in the liver stage [31]. In contrast, the *uis4* (upregulated in infectious sporozoites) gene is specifically upregulated in infectious sporozoites, those resident in the salivary glands of the mosquitoes and named accordingly [32–34]. Lastly, the *lisp2* gene, encoding the liver stage-specific protein 2, is expressed exclusively during late liver stage development prior to formation of infectious merozoites, the parasite forms infecting red blood cells [35].

ZFN-mediated DSB requires binding of two ZFNs to their respective 9–18 nucleotide long motif, followed by the dimerization of the FokI endonuclease, which then executes the DSB. We first generated a parasite line expressing two well-characterised ZFNs (ZFNL and ZFNR) shown to target the gene encoding the enhanced green fluorescent protein (eGFP) [36]. In order to express the nucleases in the sporozoite stage, one of the ZFNs was placed under the control of the *csp* promoter, and the other under the control of the *trap* promoter (Fig. 1b; Additional file 1). In addition to both ZFNs the transfection plasmid also contained the gene encoding the human dehydrofolate reductase (hDHFR) fused to *egfp.* Both are under the control of the *ef1*α promoter conferring resistance to pyrimethamine and thus allowing selection for transgenic parasites at the blood stage; the in-frame fusion of *egfp* to *hdhfr* provides the genomic sequence targeted by the ZFNs. The plasmid was integrated by double homologous crossover into chromosome 12 of the *P. berghei* strain ANKA, where integration of plasmids was shown not to interfere with neighbouring gene function and DSB would result in the loss of many genes [37, 38]. The resulting parasite line is here referred to as SpZFN. We expected that this would lead to ZFN expression during sporozoite formation, thus causing a DSB in the *egfp* gene and consequently in chromosome 12.

We also generated another parasite line expressing the two ZFNs from the liver stage-specific *lisp2* promoter, termed LsZFN (Fig. 1b; Additional file 1). Here, both *zfn* genes were separated by the self-cleaving 2A skip peptide from *Thosea asigna* virus, which has been shown to lead to efficient self-cleavage resulting in expression of two genes in *P. falciparum* [28, 39]. We expected that the ZFNs would be expressed in this parasite line during late liver stage, leading to DSBs prior to the formation of red blood cell-infecting merozoites.

We envisaged three possible outcomes following the induced DSB (Fig. 1c). If a template of the original intact locus is present in the nucleus, repair by HR could occur, leading to parasites without genomic change. This, however, would not only reconstitute the original *egfp* gene but also the binding sites of the ZFNs and thus likely lead to repeated DSBs. If no homologous repair template is available, the DSB would lead to parasites that lose a large proportion of the chromosome 12 arm during cell division and subsequent cellularisation to mature sporozoites or liver stage merozoites. In our case this would lead to the loss of 847 kb and 231 annotated genes. Of these, gene disruption has been attempted for 19 genes in *P. berghei.* Gene disruption was unsuccessful with ten of these genes, suggesting that they are essential in the blood stage (http://www.pberghei.eu). Our ZFN-parasites would, therefore, likely not be viable, and fail to establish blood-stage infections following injection of salivary gland-derived sporozoites (Fig. 1a). With a lack of NHEJ proteins, parasites might be able to repair the DSB using alternative DNA repair mechanisms such as microhomology-mediated end joining (MMEJ; Fig. 1c); this has, however, never been observed in *P. berghei*.

### Sterile protection through ZFN-mediated DSBs

After transfection of blood stage parasites and generation of clonal lines that completed the asexual cycle normally, we infected mosquitoes and challenged C57BL/6 mice with various doses of sporozoites of both SpZFN and LsZFN by intravenous (i.v.) injection (Table 1). All four mice challenged with LsZFN, and 11 out of 43 mice challenged with SpZFN, developed blood-stage parasitaemia. Only mice that did not develop a blood stage infection were subsequently re-infected with SpZFN parasites following a classic prime-two boost scheme and challenged with WT sporozoites. All fully immunised mice remained blood stage-negative after challenge with 10,000 ANKA WT sporozoites (Table 2). This shows that immunisations using sporozoites attenuated through ZFN-mediated DSBs in the sporozoite are possible and promote sterile protection. However, these data also showed that timing of the ZFN-mediated DSBs is crucial to prevent breakthrough infections.

**Table 1:** Summary of all mice challenged with sporozoites

Parasite line	Sporozoite dose injected i.v.	Infected mice/total mice	Sporozoite-induced breakthrough parasites
WT	10,000	4/4	
WT	10,000	4/4	
WT	10,000	4/4	
SpZFN	10,000	0/4	
SpZFN	25,000	1/10	SpZFN SI 1
SpZFN	25,000	3/9	SPZFN SI 2–4
SpZFN	500,000	4/4	SpZFN SI 5–8
SpZFN	25,000	3/16	SpZFN SI 9–11
LsZFN	10,000	4/4	LsZFN SI 1–4
Sp2ZFN	25,000	0/4	
Sp2ZFN	10,000	0/4	
Ls2ZFN	25,000	5/12	Ls2ZFN SI 1–5
Ls2ZFN	250,000	4/8	LsZFN SI 6–9
Ls2ZFN	1,000,000	2/4	
Uis4ZFN	25,000	0/8	
Uis4ZFN	250,000	3/8	Uis4ZFN SI 1–3
TrapZFN	25,000	2/4	TrapZFN SI 1–2
TrapZFN	250,000	0/4	
SpZFN SI 2	10,000	8/8	

*SI* sporozoite induced

**Table 2:** Protection of immunised mice against *P. berghei* ANKA WT challenge

Parasite line	Sporozoite dose i.v. for immunisation	Number of boosts	Mice positive after WT challenge	Prepatency after challenge (days)
SpZFN	10,000	2	0/4	NA
SpZFN	25,000	2	0/18	NA
Sp2ZFN	25,000	2	2/4	5.5
Sp2ZFN	10,000	2	1/4	7
Ls2ZFN	25,000	2	0/7	NA
Ls2ZFN	250,000	0	4/4	5.5

### Detection of genetic variation in parasites surviving ZFN-mediated DSBs

The 15 parasite populations arising from the breakthrough blood-stage infections were referred to as "sporozoite induced" (SI) and given a respective number (SpZFN SI 1–11, LsZFN SI 1–4). We first analysed these parasites for eGFP expression using standard fluorescence microscopy with the aim to understand why 25 % of infected SpZFN mice (11/43) and all LsZFN mice had suffered from these blood-stage infections. We observed a total of ten eGFP-expressing parasites (SpZFN SI 5–7, 9–11 and LsZFN SI 1–4), four non-fluorescent parasites (SpZFN SI 1–4) as well as one with a mixed phenotype (SpZFN SI 8) with half the parasites fluorescent and the other half not.

**Fig. 2:**
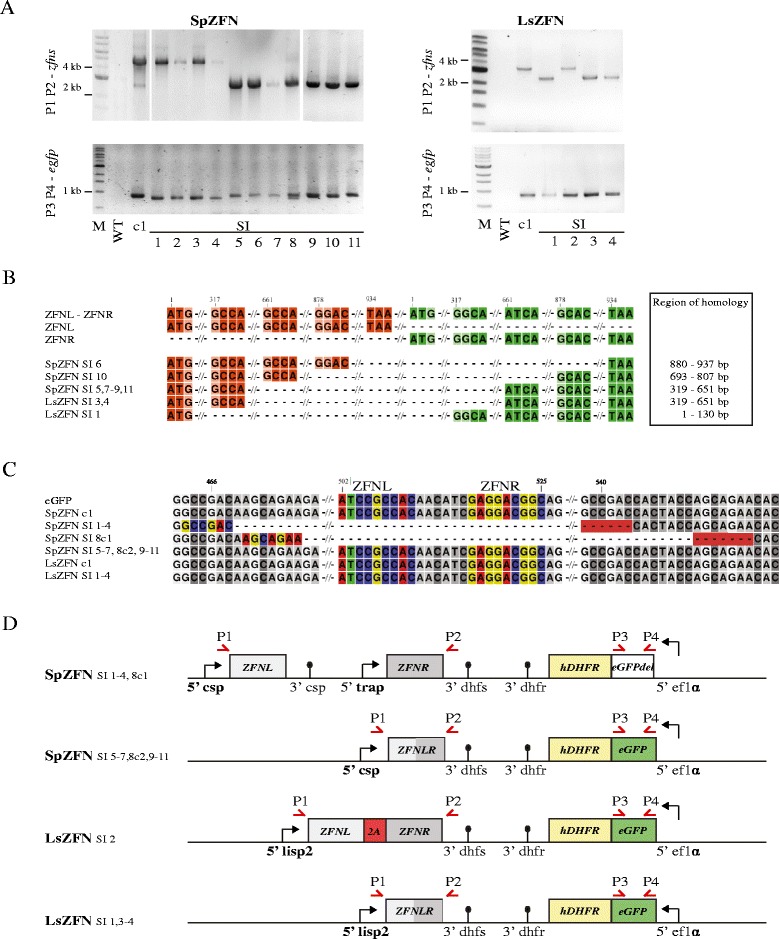
Genotype analysis of parasites surviving ZFN-induced DSB. **a** PCR analysis of the *zfn* (*P1 P2*) and *egfp* (*P3 P4*) loci from genomic DNA of blood-stage parasites. The generated transgenic clone (*c1*) and parasite populations from mice positive after sporozoite challenge were analyzed. Expected sizes of the PCR products are 4184 bp for SpZFN, 3475 bp for LsZFN and 837 bp for *egfp*. The PCR product size from the ZFN locus was smaller in SpZFN SI 5–11 and LsZFN SI 1, 3 and 4. The *egfp* product was slightly smaller in SpZFN SI 1–4 and two products are observed in SpZFN SI 8. **b** Schematic alignment of the genomic sequences obtained from all *zfn* loci with size varying from original clones. The boundaries of homology regions used for gene copy number reduction are depicted for both *zfnL* and *zfnR*. The range of perfect homology used for recombination is indicated. **c** Alignment of the sequenced *egfp* gene of all parasite lines. Binding sites of ZFNs are coloured in the first sequence and all others if present. Microhomology regions implicated in repair are highlighted in colour and with a *red background* in those sequences that have undergone repair. Note that SpZFN SI 8 was a mixed population that differed in the *egfp* gene from the other populations that underwent repair. **d** Overview of the detected genomic changes of all SI parasites. Note that all have either a modification of the *zfn* locus or of the *egfp* gene with the exception of LsZFN SI 2, which survived without any genetic changes. Positions of primers used for PCR are indicated

We next genotyped these breakthrough parasites by PCR and sequencing of the *egfp* gene as well as the locus spanning both *zfn* genes (Fig. 2). We found that PCR across both *zfn* genes from the genomic DNA (gDNA) of blood-stage SpZFN resulted in an amplicon of the expected full-length (pre-DSB) size of 4184 bp, but also detected an additional, shorter PCR product. For SpZFN SI 1–4 only the full-length product was observed; SpZFN SI 5–11, on the other hand, exclusively amplified the smaller product. Sequencing of this amplicon showed that all these parasite lines had reduced their *zfn* copy number resulting in a hybrid between *zfnL* and *zfnR* using different regions of perfect homology between the two genes, ranging from 57–333 bp (Fig. 2b). As ZFNL and ZFNR utilize two slightly mutated FokI domains to optimise formation of heterodimers and avoid formation of homodimers [40], reduction to a single *zfn* renders them non-functional, independent of their DNA binding motifs. We observed the same result for LsZFN SI 1, 3 and 4 as well.

All SpZFN parasites that retained both *zfn* genes in the genome showed a modification in the *egfp* gene, resulting in the loss of the binding sites of both ZFNs. SpZFN SI 1–4 all presented the same 75-bp deletion, originally flanked by the 6-bp microhomology region GCCGAC. One subpopulation of SpZFN SI 8 (c1) had lost 81 bp that were originally flanked by the 7-bp region AGGAGAA. Since only 11 out of 43 mice challenged with SpZFN developed blood-stage parasitaemia, we postulate that all but SpZFN SI 8 are clonal populations. All of these have either produced a single hybrid *zfn* or carry a deletion in the *egfp* gene covering the ZFN binding sites, thus preventing DSBs to occur.

The sequencing of *egfp* and *zfn* loci was consistent with our fluorescence microscopy observations: those populations that had a single recombined *zfn* copy retained an intact *egfp* and were fluorescent, while all non-fluorescent parasite lines had gone through recombination events within the *egfp* gene and retained an intact *zfn* locus.

These PCR data also showed that the *gfp* locus was intact in the parental clones, i.e. those parasites that were transmitted to the mosquitoes. However, reduction (as well as expansion) of *zfn* copy number from two to one on the other hand had already occurred at a low frequency during asexual growth of the parasite and could be detected in the SpZFN but not the LsZFN clone. This phenomenon was observed in the past with reversion of genetic modifications based on a single crossover in *P. berghei* that introduces homologous regions in close proximity within a few thousand base pairs [41]. The same mechanism is routinely used for genetic modification of malaria parasites during the removal of a negative selection marker of plasmids already integrated by single or double crossover in *P. falciparum* and *P. berghei*, respectively. It was also found that copy number variations in resistance loci most likely occur using non-allelic homologous replication or single strand annealing [23, 42].

LsZFN SI 2 had no modification in the *zfn* and *egfp* genes, suggesting that the expression of the ZFNs late during the liver stage might leave insufficient time for an efficient DSB to be produced before merozoite formation. Thus, merozoites could form that had not undergone a DSB. Alternatively, excess templates for continuous HR might be present in the liver stage due to the many copies available within one nucleus. Sequencing of the *lisp2* promoter region confirmed no mutations [35].

To test if SI parasites suffered from reduced fitness, we used SpZFN SI 2 to re-infect mice and mosquitoes and analysed its growth and infectivity across the life cycle in comparison with WT parasites (Additional file 2; Tables 1 and 3). After i.v. injection of 10,000 sporozoites as well as mosquito infection by bite, SpZFN SI 2 caused comparable blood-stage growth and experimental cerebral malaria as ANKA WT parasites. This revealed no difference and hence suggests that expression of the ZFNs does not impede parasite fitness.

**Table 3:** Infectivity of parasite strains in *Anopheles stephensi*

Parasite line	Midgut sporozoites/mosquito	Salivary gland sporozoites/mosquito	Salivary gland sporozoites/midgut sporozoites	Number of infected mosquitoes analysed
WT	111,000	21,000	0.19	30
SpZFN	84,000	12,000	0.14	18
LsZFN	157,000	29,000	0.18	22
Sp2ZFN	50,000	2500	0.05	30
Ls2ZFN	181,000	22,000	0.12	30
Uis4ZFN	5300	1700	0.32	14
TrapZFN	55,000	25,000	0.46	11
SpZFN SI 2	17,000	8300	0.49	30

### Improved second generation of ZFN parasites to reduce breakthrough rates

**Fig. 3:**
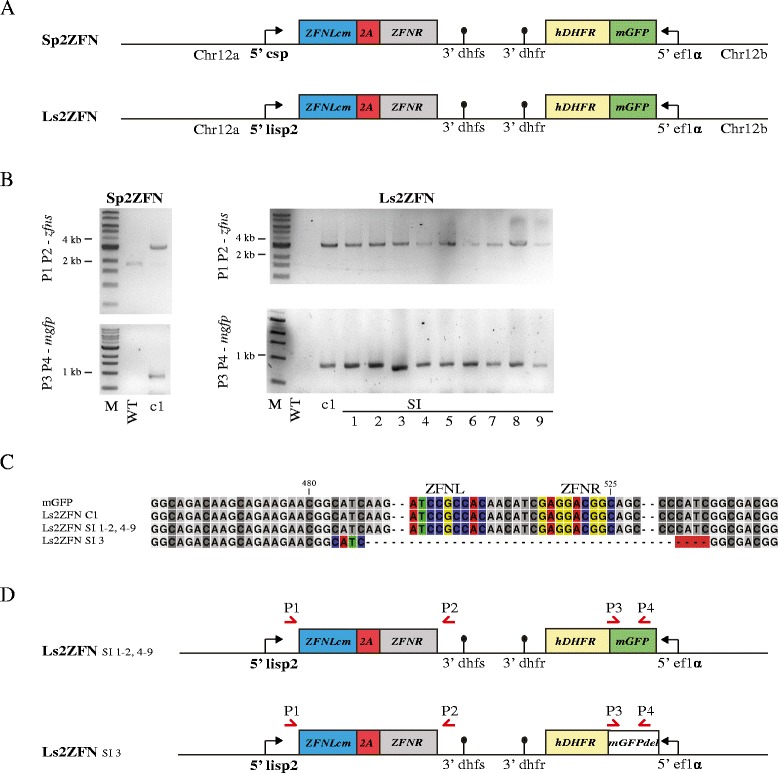
Improved second generation of ZFN parasites and genotyping of resultant SI populations. **a** Design of Sp2ZFN and Ls2ZFN. *zfnLcm* (*blue*) is a codon-modified version of *zfnL* to maximise codon difference between *zfnL* and *zfnR* (*grey*). Sp2ZFN and Ls2ZFN drive expression of both ZFNs fused by the 2A skip peptide (*red*) from a single promoter. The *mgfp* (*green*) is a codon-modified version of *egfp* that carries a silent mutation within the most frequently observed microhomology in SpZFN SI (Fig. 2c). **b** PCR amplicons of *ZFN* loci and the *mgfp* gene are shown for Sp2ZFN c1 as well as Ls2ZFN c1 and LsZFN SI 1–9. Expected sizes are 3091 bp and 3479 bp for *ZFN* loci in Sp2ZFN and Ls2ZFN, respectively. Expected size of *mgfp* is 837 bp. Only Ls2ZFN SI 3 showed a slightly smaller size for *mgfp*. **c** Alignment of all *mgfp* genes. Only Ls2ZFN SI 3 showed a deletion of 81 bp. Binding sites of ZFNs are shown in colour if present. The microhomology region of 4 bp implicated in repair is highlighted in colour and with a *red background*. **d** Overview of the genomic loci found in all Ls2ZFN SI parasites. Only Ls2ZFN SI 3 is changed in respect to the original clone Ls2ZFN c1. No SI parasites were observed for Sp2ZFN

The experiments described above suggest that the skip peptide works in *P. berghei* as in *P. falciparum*, thus allowing expression of both ZFNs from a single promoter. They also clearly imply that modification of the coding sequence of one of the two *zfn* genes is required to maintain both nucleases in the genome and prevent HR between the two genes. Based on these findings of the first generation of ZFN-expressing parasites, we designed two new parasite lines Sp2ZFN and Ls2ZFN (Fig. 3a). Those expressed both ZFNs under the control of the *csp* or *lisp2* promoter, respectively, separated by the 2A skip peptide. To avoid HR between both *zfn* genes, we codon-modified *zfnL* to *zfnLcm* to have the lowest possible homology with *zfnR*. This was accomplished by first codon-optimizing *zfnL* for *P. berghei* codon usage [43, 44] and then manually changing all codons still identical to *zfnR* whenever possible (Additional file 3). Additionally, we introduced a silent point mutation within the 6-bp homology of *egfp* that had been utilised to resolve the DSB in SpZFN SI 1–4, generating *mgfp*.

While both parasite lines showed no phenotypic difference to WT parasites prior to sporozoite formation, sporozoite numbers of Sp2ZFN were strongly reduced in the salivary gland (Table 3). This limited the number of experiments we could perform and also rendered this parasite line non-practical for use as an attenuated parasite line in large-scale vaccination experiments. Additionally, not all mice immunised with Sp2ZFN showed sterile protection after challenge with 10,000 *P. berghei* ANKA WT sporozoites (Table 2), which is in agreement with the early liver stage arrest we observed in an in vitro liver stage development assay (Additional file 4). Challenge of C57BL/6 mice with Sp2ZFN and Ls2ZFN parasites resulted in either a block of parasite development (all Sp2ZFN and some Ls2ZFN) or the emergence of blood-stage parasites. The latter populations were termed Ls2ZFN SI 1–9. Of the parasites derived from Ls2ZFN challenge, Ls2ZFN SI 1, 2, 4–9 showed GFP expression whereas Ls2ZFN SI 3 was non-fluorescent. We genotyped all parasite lines by PCR and sequencing (Fig. 3) and found no modifications of the ZFN locus, validating the improved genetic stability resulting from our codon-modification approach. In Ls2ZFN SI 3 parasites, however, we observed an 81-bp deletion within *mgfp* that includes the ZFN binding sites, originally flanked by the 4-bp microhomology CATC. All other SI blood-stage parasites showed no genetic modification and most likely escaped DSB by HR or non-timely ZFN expression.

### Optimisation of ZFN expression timing

**Fig. 4:**
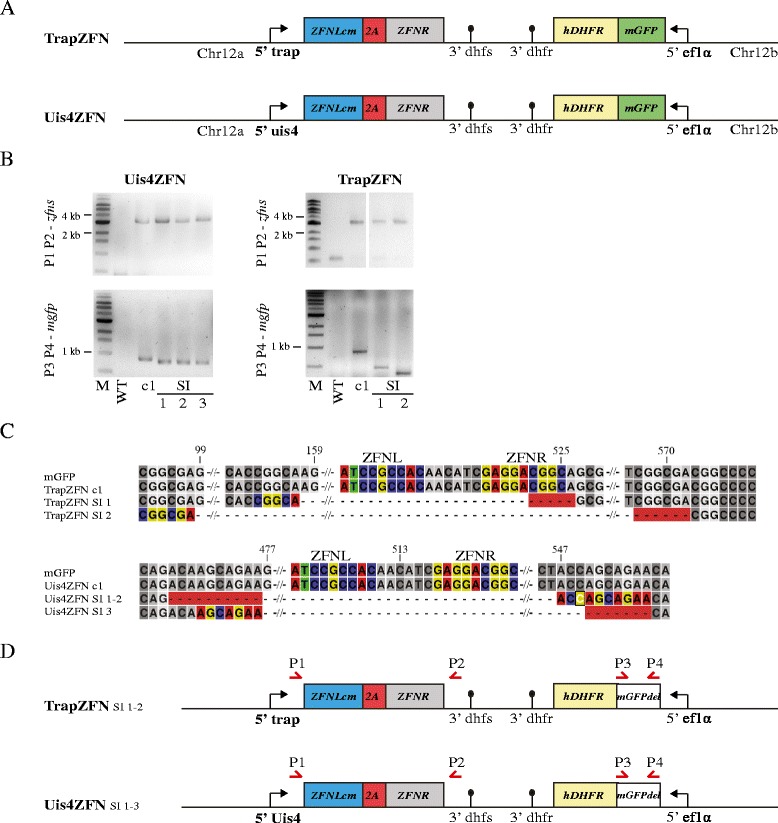
ZFN parasites with optimised expression timing and genotyping of SI populations. **a** Design of TrapZFN and Uis4ZFN. Both parasite lines express ZFNs fused with a 2A skip peptide under control of the respective promoters of *trap* and *uis4*. **b** PCR products of the *zfn* loci and the *mgfp* gene are shown for parental clones Uis4ZFN c1 and Uis4ZFN SI 1–3 and TrapZFN c1 as well as TrapZFN SI 1–2. The expected size of the *zfn* genes was 3303 bp for Uis4ZFN, 3321 bp for TrapZFN and 837 bp for *mgfp*. The ZFN loci were all identical in size, whereas Uis4ZFN SI 1–3 and TrapZFN SI 1–2 showed shorter PCR products for *mgfp* of varying sizes. **c** Alignment of all *mgfp* genes. TrapZFN SI 1 had a deletion of 369 bp; in TrapZFN SI 2 474 bp were deleted. Binding sites of ZFNs are coloured and the microhomology regions implicated in repair of 6 and 7 bp, respectively, are highlighted in colour and with a *red background*. Both Uis4ZFN SI 1–2 and SI 3 had a deletion of the same 81 bp. Microhomology regions implicated in repair are 10 bp, including one mismatch highlighted in *white* for SI 1–2 and 7 bp for SI 3. **d** Overview of the genomic loci found in both TrapZFN SI 1–2 and Uis4ZFN SI 1–3. Both TrapZFN SI parasites have big deletions in the *mgfp* gene with respect to the original clone TrapZFN c1; Uis4ZFN SI parasites show a small deletion in the *mgfp* gene

The experiments with the second generation of parasites suggested that timing of ZFN expression was either too early (Sp2ZFN) or too late (Ls2ZFN). We therefore aimed to identify optimal promoters that are driving expression either late during sporozoite formation or once sporozoites are formed. In SpZFN, the *trap* promoter mainly determined the timing of DSB, as it becomes active after the *csp* promoter. We thus argued that using this promoter instead of the stronger and earlier *csp* promoter should result in more viable sporozoites and hence generated the respective parasites, termed TrapZFN. We also speculated that a slightly later induction of DSB within the salivary gland might be even more beneficial, as all sporozoites contain only a single copy of the genome. Additionally, DNA repair proteins might be down-regulated in the sporozoite stage compared with the actively dividing oocyst. We thus selected the *uis4* promoter, which is active only once sporozoites are within the salivary gland. The generated parasite line using this promoter was called Uis4ZFN (Fig. 4a). As expected, both parasite lines showed an increased salivary gland to midgut ratio compared with Sp2ZFN (Table 3) and an intermediate development in HepG2 cells compared with Sp2ZFN and Ls2ZFN (Additional file 4).

We then injected mice with 25,000 and 250,0000 sporozoites of each of the two lines. Eight mice injected with a total of 1.1 million TrapZFN sporozoites resulted in TrapZFN SI 1–2, whereas 16 mice injected with 2.2 million sporozoites of Uis4ZFN resulted in Uis4ZFN SI 1–3 (Table 1), all non-fluorescent. Genotyping of the SI parasite TrapZFN and Uis4ZFN populations showed that they contained the unmodified ZFN locus (Fig. 4b), again confirming that the use of codon-modified ZFNs prevented unwanted recombination events. However, TrapZFN SI 1 had a deletion of 369 bp within *mgfp* originally flanked by the microhomology sequence CGGCA, and TrapZFN SI 2 showed a loss of 474 bp originally flanked by the sequence CGGCGA (Fig. 4b, c; Additional file 5). Genotyping of Uis4ZFN SI 1–3 revealed the same 81-bp deletion for all three clones flanked by 10 bp of microhomology; however, this sequence contained a single mismatch (Fig. 4b, c; Additional file 5). This curious result suggests that MMEJ resulted in a single mismatch that was tolerated and gave rise to two populations during the following mitotic divisions leading to sporozoite formation. As these three clones have been isolated from three individual mice, it is more likely that the DSB occurred in the oocyst before sporozoite formation and not as three independent DSBs in salivary gland sporozoites being repaired with the same 10 bp of microhomology. Thus, we expect that at least in one oocyst, the *uis4* promoter was active and not, as expected, silent until the salivary gland sporozoite stage.

### Sequence determinants for MMEJ

Having identified seven different MMEJ events in 11 parasite lines we tried to identify whether they shared potential requirements for homology regions utilized during DNA repair (Additional file 5). The average GC content of the homology regions used for repair was 68 % compared with 62 % within the entire *egfp*. We would expect that the amount and length of possible homology regions might increase within the AT-rich intergenic regions in *Plasmodium* but that high GC content should favour stronger binding. A computationally scrambled DNA sequence of 1000 bp with a GC content of 20 % contained 91 homology regions of at least 8 bp, whereas the 795 bp long open reading frame of *egfp* still contained 77 homology regions of at least 7 bp in length. This implies that the parasite can potentially repair a DSB at every genomic location, but frequencies of repair are low and depend on the life stage timing of DSB.

### Analysis of chromosomal integrity of parasites failing to repair the DSB

**Fig. 5:**
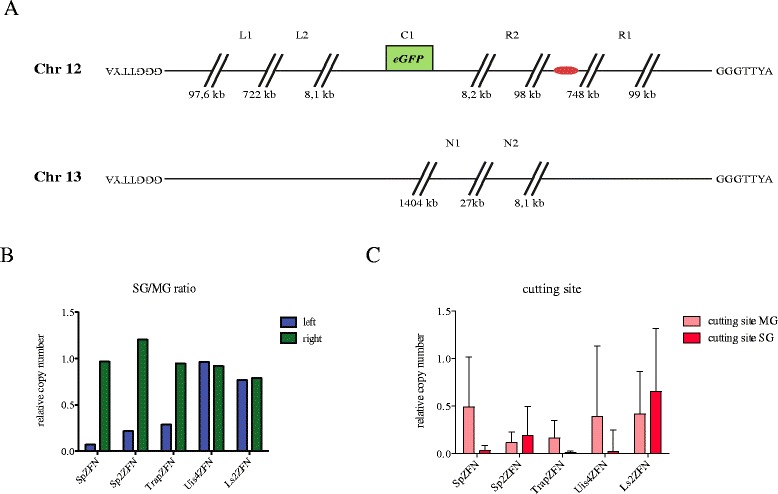
Copy number analysis by qPCR on genomic DNA. **a** Schematic overview of chromosomes 12 and 13. The centromere of chromosome 12 is shown in *red*. Binding sites for primer pairs used for qPCR are shown. Primer pair C1 amplifies the product over the cutting site of the ZFNs, while primer pairs L1 and R1 bind approximately 100 kb away from the telomeres on the left and right arm of chromosome 12, respectively. L2 and R2 bind around 8 kb away from the cutting site. N1 and N2 bind on the ‘control’ chromosome 13 and are used for normalization. **b** The ratio of the relative copy number of amplicons from both sides of the break point on chromosome 12 (L1 and L2 on left of break; R1 and R2 on right of break) is shown for parasites isolated from mosquito salivary glands (*SG*) compared with parasites isolated from midguts (*MG*). The copy number of the left side of chromosome 12 is strongly reduced in the SG sample for parasites expressing ZFNs in the midgut, whereas the right side is not affected. All individual values, including errors, are shown in Additional file 6. **c** The relative copy number of PCR products amplified over the break point shown for genomic DNA isolated from midgut oocysts (*MG*) and from salivary gland sporozoites (*SG*). Note the near absence of product in salivary glands from parasites where ZFNs are expressed before (*SpZFN*, *TrapZFN*) or during (*Uis4ZFN*) sporozoite entry into salivary glands. Positive and negative error is calculated from standard error of the mean from technical duplicates

So far, all our analysis concerned the few parasites that managed to survive their experimentally designated fate. However, we also aimed to analyse the main populations of parasites that failed to resolve the DSB and did not progress through the life cycle. In order to do so we opted for quantitative PCR (qPCR) analysis of genes located at either side of the DSB. This approach would also allow us to effectively quantify the efficiency of ZFN activity in the parasite. We extracted genomic DNA from original blood-stage parasites (BS), infected mosquito midguts 10 days post-infection (MG) and from infected salivary glands 17 days post-infection (SG). We designed primes to amplify short (131–216 bp) DNA fragments on either side of the *egfp* gene as well as spanning the *egfp* ZFN binding site (Fig. 5a). As a control we chose primer pairs to amplify genes from chromosome 13.

Measurement of the relative gene copy number showed the presence of all DNA fragments within all parasite lines (Additional file 6). We combined the data for the left part of chromosome 12 lacking the centromere (with respect to the cutting site) and the right part of chromosome 12 that contains the centromere. The ratio of SG to MG shows the relative copy number of both sides that is retained in salivary gland sporozoites (Fig. 5b). Parasite lines expressing the ZFNs within the oocyst (SpZFN, Sp2ZFN and TrapZFN) showed a strong reduction in the relative copy number of the left part of chromosome 12 but not the right part. Uis4ZFN as well as Ls2ZFN, on the other hand, retained both parts of chromosome 12 in the salivary gland. This was independent of the occurrence of the DSB, as revealed by the qPCR signal across the cutting site. This PCR product can only be produced if the *egfp* is still uncut; it was already very low for Uis4ZFN in the salivary gland but still high in Ls2ZFN parasites, which did not express the ZFNs yet (Fig. 5c). These results indicate that it is not the DSB itself that causes the developmental defect and that cell division is not arrested after the DSB. They also suggest that as soon as nuclear division takes place the centromere-devoid part of the chromosome is lost.

## Discussion

**Fig. 6:**
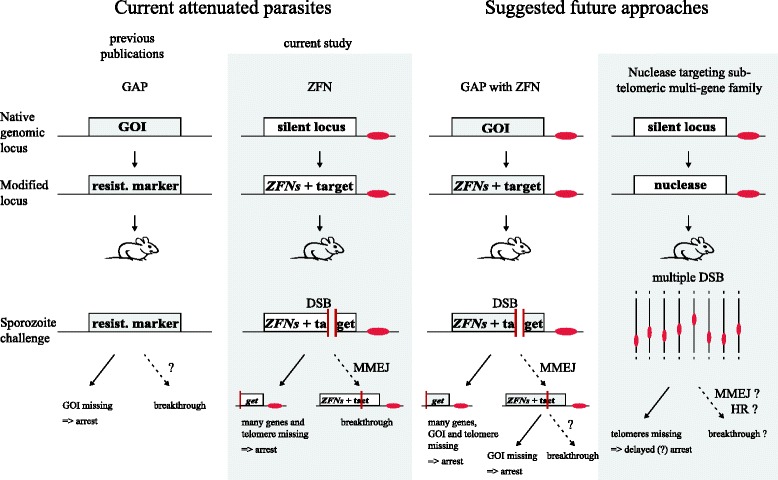
Current and potential future liver stage attenuated parasites. GAP parasites are generated by the deletion of a gene of interest (*GOI*) essential for the *Plasmodium* liver stage. While this leads to developmental arrest in the liver in most cases, rare breakthrough events are observed. The frequency depends on the GOI, and how single parasites survive is not understood. ZFN arrested parasites described in this manuscript arrest as they lose the arm of the chromosome lacking the centromere after DSB. Rare breakthrough events are the result of DSB repair via MMEJ and result in loss of the ZFN target site. Potential future approaches include the combination of GAPs with ZFNs. A single genetic modification allows for the replacement of a GOI with a ZFN construct. These parasites have to overcome both the DSB via MMEJ and the missing gene to result in a breakthrough infection. However, the localisation of the GOI on the chromosome influences the number of genes lost after DSB and thus possibly attenuation. Another potential approach is the use of a nuclease to target conserved sites in a subtelomeric multi-gene family (Additional file 7). This leads to multiple DSBs, resulting mainly in the loss of telomeres. Developmental arrest might be delayed. Potential repair can occur via MMEJ, resulting in the loss of the target site or via HR restoring the target site. Centromeres are drawn in *red*, DSBs are depicted with a gap, *dashed arrows* mark rare events

Here we show that ZFNs can be used to generate attenuated *P. berghei* parasites with a single DSB. Parasites with an unresolved DSB lose their centromere-lacking half of the broken chromosome during the next cell division, resulting in the loss of hundreds of genes. When the DSB is correctly timed, sporozoites can still invade liver cells but arrest in development and are unable to produce blood-stage infections. Immunisation with some of these parasites results in a sterile protection that is comparable to traditional GAPs. This, together with the data from all previous GAP immunisation studies, hints to the fact that parasites arresting within the liver always confer protective immunity from subsequent challenges with WT parasites. This appears independent from the cause of arrest [45], although subtle differences in protective efficacy can be observed [46]. In follow-up studies it will be interesting to observe potential differences between ZFN-mediated and classic GAP parasites, especially when ZFNs are expressed in different loci (Fig. 6).

The deletion of a single GAP candidate gene frequently results in breakthrough infections in the experimental mouse host. Although originating from a clonal population, a few mutant parasites often manage to survive in the absence of a particular protein [16]. The dearth of gene expression data for liver stages of human malaria parasites impedes the rational choice of GAP target genes. Also, transfer of experimental data from the rodent model parasites to *P. falciparum* has not been straightforward [21]. This is likely due to the nature of the liver stage. In contrast to motile stages that exhibit many essential stage-specific genes, liver stage-essential genes can be broadly grouped into two subsets. Genes involved in growth and cell division are mainly shared with the blood stage and thus are not suitable for GAPs as their deletion is often not possible. Other genes are involved in host–pathogen interaction. These genes are part of the evolutionary battle between the parasite and its host cell. Here, a shifted balance in favour of the host cell after gene deletion might be partially reversed if additional diseases or environmental factors affect the host, thus causing breakthrough infections. This is not an issue in ZFN-induced DSB as survival will only depend on the repair capabilities of the parasites and not be affected by the host cell.

For traditional GAPs, breakthrough infections are not genetically tractable as they are not a product of genetic modification. In contrast, here we could analyse the reason for parasite survival in our ZFN-induced DSB-mediated GAPs. We found two ways by which parasites escaped their designated fate. The first utilized an inherent property in the design of ZFNs, which are long stretches of identical coding regions shared by the two ZFNs. Parasites used these homologies to loop out the region in-between the ZFNs during blood-stage growth. We assume that this might be induced by a random DSB [23]. HR utilising close/adjacent homologous regions of several hundred base pairs in length has been observed sporadically [47]. Indeed, this mechanism is routinely relied upon in negative selection approaches that allow sequential genetic modification in the absence of multiple selection markers as only two positive selection markers are used in *P. berghei* research [42]. Our work addresses the molecular requirements behind the phenomenon of recombination in *P. berghei*. We found recombination events based on large-sized homology regions in the ZFN coding regions akin to experimental negative selection. This occurred more frequently in LsZFN where the *zfn* genes are closer to each other than in SpZFN. This suggests that the distance between homology regions might increase the likelihood of recombination. In the case of SpZFN, to generate sporozoites with only one *zfn*, both parasites must have reduced the locus before they form the zygote since the parasite will remain diploid until sporozoite formation (Fig. 1a). In the case of LsZFN, only the single haploid genome copy of the sporozoite invading the liver cell must have had its copy number reduced before invasion. We also showed that such recombination can be prevented by codon modifications to avoid any homology within the recombination-prone sequence (Additional file 3). These observations also led us to consider why there have been no reports on the successful use of TALENs (transcription activator-like effector nucleases) in *Plasmodium* until now. Given that the central repeat domain contains 18–20 repeats coding for 33–34 amino acids each and only differing by a few base pairs, we would suggest that TALENs might reduce their number of repeats within the parasite [48].

More importantly, parasites could survive a DSB by repair that was found to proceed with as little as 4-bp homologies. In the seven different repair products we observed, we found 4–10-bp homologies with up to one mismatch, deletions of 75–474 bp, and no insertions (Additional file 5). These repair events have the characteristics of MMEJ, which is defined by homology of 5–25 bp and the deletion of the previously flanked sequence [49]. These events are most likely extremely rare; an estimation based on the number of SI clones observed during the immunisation protocol suggests about one survivor out of 50,000–700,000 initially injected parasites. Comparison of these breakthrough rates within the ZFN parasites and with published GAPs is not straightforward, mainly due to sporozoite doses used in the various studies. In our limited sample size of breakthrough mice we found a very weak correlation of chance for breakthrough with the number of parasites injected for Ls2ZFN, a good correlation for Uis4ZFN and an inverse correlation for TrapZFN (Table 1). This would predict that the injection of, for example, 100 mice with 25,000 sporozoites each would result in a different number of breakthrough events than the injection of 5 mice with 500,000 sporozoites each. Published breakthrough rates range between one out of two mice for *uis4(−)* [50] and non at all for *fabb/f(−)* [17]. This suggests that our ZFN parasites fall within that range. Without the selection of mutants that lose the ZFN cutting site, this small population of mutants would not have been observable. As the numbers of breakthrough infections varied strongly between parasite strains and experiments, we expect them to be influenced by the exact timing of the DSB. Of the parasite lines tested, we would favour Uis4ZFN and TrapZFN for future experiments. While Uis4ZFN has the advantage that the main expression of the *zfn* genes only starts in the salivary gland, TrapZFN stops *zfn* expression in the liver stage, which could be favourable in terms of application safety if this system is applied to *P. falciparum.*

Of more than 12.5 million sporozoites injected in this study, no parasite managed to survive with a mutation in *zfn* genes or with the DSB once it occurred and part of chromosome 12 was lost. The latter parasites did arrest most likely due to loss of multiple genes as well as chromosomal instability due to telomere loss.

Another study in *P. falciparum*, utilizing a Sce-I-induced DSB, also observed end joining repair products in the absence of homologous templates [27]. In contrast to our findings, Kirkman et al. [27] observed repair independent of obvious microhomologies and a maximal loss of 5 bp in combination with a 2 bp insertion. While this might hint at a different mechanism of repair, the observed rate of repair was similarly inefficient to the one observed in our experiments. The different outcomes of repair might not, therefore, necessarily rely on different mechanisms of DNA repair between *P. berghei* and *P. falciparum* but could be due to the different ends created by the Sce-I and ZFNs. Also, the observed repair occurs in different stages of the life cycle, as the study by Kirkman and colleagues was performed in the blood stage. Thus, we cannot exclude that the different stages might influence the expression of proteins involved in DNA repair pathways. In this respect, it is also worth noting that microhomology pairing in blood-stage parasites occurs at 37 °C but only at 21 °C in mosquitoes. Possible differences could be tested by expressing Sce-I in *P. berghei* sporozoites in a similar fashion as we expressed the ZFNs.

MMEJ has been shown to require six key proteins in mammals: MRE11, NBS1, LIGASE III, XRCC1, FEN1 and PARP1 [51]. Of these, only MRE11, XRCC1 and FEN1 have been identified in *Plasmodium*, and MRE11 has recently been characterised in *P. falciparum* [52]. Of those missing, DNA ligase III seems to be replaceable by DNA ligase I [53] and PARP1 is only present in the related parasite *T. gondii*, supporting its suggested role to compete with Ku for choice of repair pathway [54]. NBS1 is only present in eukaryota and seems to be the least conserved part or the MRN complex, which otherwise consists of MRE11 and RAD50, which are also conserved in bacteria and archaea [55]. NBS1 shows no sequence homology with its functional homolog in *Saccharomyces cerevisiae*, XRS2. This makes identification of a homolog in *Plasmodium* via homology searches unlikely. Very recently, the human polymerase θ was identified to promote alternative NHEJ and suppress homology-dependent repair [56, 57]. We identified a potential homolog in *P. berghei* [GenBank:CDS50785.1; PBANKA_134600]. If any of those factors are essential for MMEJ but not for life cycle progression in *Plasmodium*, their deletion could be combined with the ZFN parasites to abrogate MMEJ repair after DSB.

The use of ZFN allows the simultaneous deletion of tens to hundreds of genes for the generation of attenuated parasites. Our work is a proof-of-principle using only a single cleavage site within the entire genome. This suggests that the inclusion of several more cleavage sites should prevent any breakthrough infections. Such additional targets could either be introduced throughout the genome experimentally (as shown in this work) or rely on endogenous sequences. The latter approach could most easily be established by targeting conserved genomic regions that are present in multi-gene families, such as *bir* in *P. berghei*, *yir* in *P. yoelii*, or *var* in *P. falciparum*. While this could be achieved with ZFNs, it could also be tested by utilising CAS9 [58], as shown for multi-copy genes in *Trypansoma cruzi*, the causative agent of Chagas disease [59]. These approaches should result in telomere loss and slow decline of the parasite population instead of immediate arrest, which could be beneficial for the generation of attenuated parasites. Deletion of *P. berghei* telomere reverse transcriptase has been performed and resulted in a delayed death phenotype [60]. We found up to 104 targets with a single target site in the *bir* gene family, 522 in the *yir* gene family and up to 39 for the *var* gene family (Additional file 7). This strategy has two potential Achilles heels: a single mutation in the nuclease could result in a loss of function resulting in breakthrough; additionally, multiple target sites will not all be cut at once, so besides MMEJ the parasite could also utilize HR to repair the DSB. Ultimately, genetically attenuated parasites will have to be generated from a combination of multiple “arresting phenotypes”. This is the current rationale for deleting multiple genes involved in different parasitic mechanisms or pathways [22, 61]. Hence, a nuclease-based method can easily be combined with a single gene knockout by introducing the ZFN cassette into the locus of a GAP candidate gene during the generation of that GAP parasite (Fig. 6). Since timing of expression can be easily changed for ZFN-induced DSB, it should be possible to combine multiple arrested phenotypes to arrest at the same time. Change of localisation of the DSB into the locus of a GAP candidate gene will influence the genes that are lost after the DSB. In the case of subtelomeric localisation, this could reduce the promptness of arrest, but ultimately should result in death of the parasite due to genome instability. We have summarised genetic attenuation strategies tested so far and compared them with the suggested strategies in Fig. 6.

## Conclusions

Here we probed the suitability of ZFN-induced DSBs to investigate DNA repair mechanisms and for the generation of attenuated parasite lines in a rodent malaria parasite species. Our data show clearly that *Plasmodium* can repair, at low frequency, DSBs using MMEJ. We could also show that attenuated parasite lines can be generated for use in experimental vaccination studies and provide a rationale for moving these forward towards generation of safe attenuated parasite strains for potential use in clinical studies.

## Materials and methods

### Animal work

All animal experiments were performed according to the FELASA and GV-SOLAS standard guidelines and were approved by the German authorities (Regierungspräsidium Karlsruhe). Experiments to generate parasite lines and infect mosquitoes were performed with NMRI mice and parasites involving sporozoite injections were performed with female C57BL/6 mice (both from Charles River).

### Plasmid generation

All vectors used for this study were derived from Pb237 [38]. The following modifications were made. A 565-bp upstream region of ef1α was amplified from *P. berghei* ANKA WT gDNA with P5/P6 (Additional file 8) and cloned with AgeI/ApaI to replace the promoter sequence driving the selection marker. The *hdhfr* gene was amplified from human cDNA with P7/P8 and cloned with AgeI/NheI. Next *egfp* was amplified with P9/P10 and cloned upstream in frame with *hdhfr* with AgeI to result in an *egfp-hdhfr* fusion gene (Pb238). To generate the expression box for the ZFNs, subcloning was performed in pGEM. The promoter of CSP was amplified with P11/P12 and cloned into pGEM. *zfnL* was amplified with P13/P14 and inserted with KpnI/PshAI, followed by insertion of the 3’ UTR of *csp* (P15/P16) with PshAI/SwaI. In parallel, the fragments of the 3’ UTR of *dhfs*, *zfnR* and TRAP promoter were amplified with P17/P18, P19/20 and P21/P22, respectively, cloned into pGEM with direct ligation, PshAI/KpnI and SwaI/PshAI, and cloned with EcoRV/SwaI into the first pGEM vector. The whole fragment was cloned with NotI/EvoRV into Pb238, resulting in the vector SpZFN. To generate LsZFN, the promoter of *lisp2* was amplified with P23/P24, *zfnL* was amplified with P25/P26, introducing the 2A skip peptide via P26. Both PCR products were fused with overlap extension PCR using P23/P26 and cloned into SpZFN with NotI/PshAI.

To generate *mgfp* for the following vectors, two fragments of *egfp* were amplified with P27/P28 and P29/P30, fused with overlap extension PCR P27/P30 and cloned into LsZFN with SwaI/PstI. The *zfnL* gene was codon-modified with the codon usage table from *P. berghei* (http://www.kazusa.or.jp/codon/) [43] that was applied with OPTIMIZER (http://genomes.urv.es/OPTIMIZER/) [44]. The resulting coding sequence was aligned with *zfnR* and in all identical codons a silent mutation was introduced wherever possible, resulting in an additional 21 bp changed. The resulting sequence, *zfnLcm* with fused 2A skip peptide, was ordered from GeneArt (Regensburg). The promoter of *csp/lisp2* was amplified with P31/P32 and P33/P34, cloned via NotI/HindIII into *zfnLcm* and together with *zfnLcm* cloned into LsZFN (*mgfp*) with NotI PshAI, resulting in Sp2ZFN and Ls2ZFN, respectively. To generate TrapZFN and Uis4ZFN, the respective promoter regions were amplified with P35/P36 and P37/P38, and cloned with NotI/NdeI into Sp2ZFN.

### Parasite transfection and sporozoite generation

Transfection was essentially performed as published [62]. All vectors were linearised with PvuI prior to transfection and integrated into chromosome 12 between bases 846,483 and 847,711 using two homology regions, Chr12a and Chr12b, with lengths of 481 and 431 bp, respectively. All parasite lines were generated by a single transfection. PCR to confirm correct integration was performed after transfection and after limiting dilution cloning (Additional file 1). *Anopheles stephensi* mosquitoes were infected with clonal lines as described previously [63].

### Immunisation

Mice were immunised with SpZFN, Sp2ZFN and Ls2ZFN sporozoites. C57BL/6 mice were injected i.v. with sporozoites in RPMI medium. Immunisation was performed with three injections, one prime and two boosts. The time after injection until the next boost and the challenge with ANKA WT sporozoites was 14, 7 and 7 days for 10,000 SpZFN sporozoites for 4 mice and with 25,000 sporozoites for 13 mice. All other immunisations were performed with gaps of 14, 7 and 14 days. Mice challenged after single dose immunisations were challenged 35 days after prime (Table 2).

### PCR analysis and sequencing

All parasites that managed to result in blood-stage parasites after blood-stage challenge (SI) were analysed with PCR. The *zfn* locus was amplified with P1(Sp/Ls/Uis4/Trap)/P2 and *egfp/mgfp* was amplified with P3/P4. Additionally, promoter sequences of LsZFN and Ls2ZFN SI parasites lacking any modification in *zfn* and *egfp/mgfp* were amplified with P53/P34 and sequenced. PCR products were purified and sequenced at GATC (Konstanz).

### Quantitative PCR

We purified gDNA from midguts 10 days post-infection (MG) and salivary glands 17 days post-infection (SG) and mixed blood stages (BS). When possible, 200,000 sporozoites were used for gDNA production. Primers for qPCR were chosen to amplify a product over the cutting site C1 (P39/P40), 8133 bp upstream of the cutting site L2 (P41/P42) and 8240 bp downstream of the cutting site R2 (P43/P44). Additional probes were designed 100 kb away from the telomere upstream L1 (P45/46) and downstream R1 (P47/P48) of the cutting site. Probes for normalisation were designed for chromosome 13, N1 (P49/P50) and N2 (P51/P52). qPCR was performed with SYBR Green PCR master mix (Life Technologies) on an ABI7500 thermo cycler (Applied Biosystems) with 40 cycles of 15 s denaturation at 95 °C and 1 min at 60 °C. Reactions were performed in 12.5 μl final volume in technical duplicates. Fold differences of SG and MG samples relative to BS samples were calculated according to the 2^ΔΔCT^ method, using N1 and N2 as housekeeping loci.

### Microscopy

Imaging was performed with an inverted Axiovert 200 M microscope from Zeiss (Jena). Live blood stages were imaged with a GFP filter set and differential interference contrast. Liver stages were fixed after 48 h with 4 % paraformaldehyde for 15 min, stained with α-CSP (mAB 3D11) or α-UIS4 antibody and α-GFP antibody Abfinity™ from Life Technologies. Image processing and size measurements were performed with Fiji [64].

## Additional files

Additional file 1: Fig. S1. Integration PCRs of parasites used in this study. **a** Integration of SpZFN and LsZFN. PCR with P53/P54 amplifies the whole Chr12 locus, resulting in 1448 bp in WT, 7850 bp for SpZFN and 7141 bp for LsZFN. The 5’ integration was amplified with P53/P12 or P53/P24, resulting in 1226 bp and 1610 bp for SpZFN and LsZFN, respectively. The 3’ integration was performed with P54/P55, resulting in 1181 bp. **b** Integration of Sp2ZFN and Ls2ZFN. PCR of the whole locus resulted in 6758 bp or 7145 bp for Sp2ZFN and Ls2ZFN, respectively. The 5’ integration was performed with P53/P56 or P53/P24, resulting in 1226 bp or 1610 bp for Sp2ZFN and Ls2ZFN. The 3’ integration was verified with P54/P10 (1982 bp). **c** Integration of TrapZFN and Uis4ZFN. PCR of the whole locus resulted in 6988 bp and 6970 bp for TrapZFN and Uis4ZFN, respectively. The 5’ integration was performed with P53/P22 or P53/P38, resulting in 1462 bp and 1504 bp for TrapZFN and Uis4ZFN, respectively. The 3’ integration was verified with P54/P8 (2588 bp). (PDF 1088 kb) Additional file 2: Fig. S2. Characterisation of SpZFN SI 2. **a** Liver stages of parasites in HepG2 cells 48 h after sporozoite invasion. CSP staining shows the plasma membrane of the parasite within the hepatocyte. **b** C57BL/6 mice were challenged with 10,000 sporozoites i.v. or by the bites of ten infected *A. stephensi* mosquitoes (*bb*). Peripheral blood parasitaemia was monitored by Giemsa-stained blood smears from three days post-infection. Percentage of mice that are blood stage (*BS*) parasite free and percentage of mice alive is shown over time. Parasitaemia over the course of the experiments is shown as growth curves. Control infections with SpZFN resulted in no blood stage parasitaemia. (PDF 1601 kb) Additional file 3: Fig. S3. Alignment of *zfnL* and *zfnLcm.* (PDF 1188 kb) Additional file 4: Fig. S4. Liver stages of parasites in HepG2 cells 48 h after sporozoite invasion. CSP or Uis4 staining shows the plasma membrane of the parasite within the hepatocyte. Staining with α-GFP antibody shows residual expression of mGFP-hDHFR fusion protein. Sizes of liver stages from single slice images were counted 24 h and 48 h post-sporozoite invasion. (PDF 7464 kb) Additional file 5: Fig. S5. Overview of all microhomology sequences used by SI parasites to repair DSBs. Microhomology sequences are shown in bold. Ten base pairs flanking each homology region are shown prior to the DSB break and the remaining bases in the SI parasites. The number of base pairs lost after repair is indicated. The mismatch in the microhomology sequence used for repair of Uis4ZFN SI 1–3 is highlighted in *orange*. (PDF 280 kb) Additional file 6: Fig. S6. Results of qPCR on gDNA. **a** Localisation of qPCR probes on chromosomes 12 and 13. The centromere of chromosome 12 is shown in *red*. Binding sites for primer pairs used in qPCR are shown. Primer pair C1 amplifies the product over the cutting site of the ZFNs, while primer pairs L1 and R1 bind approximately 100 kb away from the telomeres on the left and right arm of chromosome 12, respectively. L2 and R2 bind around 8 kb away from the cutting site. N1 and N2 bind on the ‘control’ chromosome 13 and are used for normalization. **b** Chromosome 12 integrity is shown for samples from salivary gland (*SG*) and midgut (*MG*). Values were normalised to N1 and N2 on chromosome 13 and to results from blood-stage gDNA amplification. Positive and negative error is calculated from standard error of the mean from technical duplicates. (PDF 350 kb) Additional file 7: Fig. S7. Map of chromosomal location of multiplex target sites using CAS9. **a** All perfect matches for the possible gRNA sequence TTATATTAGTATTCGTTATTTGG targeting 104 loci of the *P. berghei* genome in different *bir* genes and *bir* pseudogenes. Archived contigs represent sequences that have not successfully been mapped to a chromosome. **b** The same sequence targets 532 targets in the *P. yoelii* genome within 522 different *yir* and *yir* pseudogenes. **c** Possible gRNA sequence GGTAACAACACAACAGCTAGTGG matches 39 targets within 31 *var* genes in *P. falciparum*. Figures were created with Plasmodb.org [65]. (PDF 996 kb) Additional file 8: Fig. S8. A list of all primers used in this study. (PDF 396 kb)

## Abbreviations

bp: base pair
CSP: circumsporozoite protein
DSB: double-strand break
eGFP: enhanced green fluorescent protein
GAP: genetically attenuated parasite
gDNA: genomic DNA
hDHFR: human dehydrofolate reductase
HR: homologous recombination
i.v.: intravenous
MMEJ: microhomology-mediated end joining
NHEJ: non-homologous end joining
PCR: polymerase chain reaction
qPCR: quantitative polymerase chain reaction
SI: sporozoite induced
TRAP: thrombospondin-related anonymous protein
UTR: untranslated region
WT: wild type
ZFN: zinc-finger nuclease

## References

[1] WHO. World Malaria Report 2014. Geneva: WHO Press; 2014. http://www.who.int/malaria/publications/world_malaria_report_2014/report/en/. Accessed 24 July 2015.

[2] Wipasa J, Okell L, Sakkhachornphop S, Suphavilai C, Chawansuntati K, Liewsaree W (2011). Short-lived IFN-gamma effector responses, but long-lived IL-10 memory responses, to malaria in an area of low malaria endemicity. PLoS Pathog.

[3] Marsh K, Kinyanjui S (2006). Immune effector mechanisms in malaria. Parasite Immunol.

[4] Cowman AF, Morry MJ, Biggs BA, Cross GA, Foote SJ (1988). Amino acid changes linked to pyrimethamine resistance in the dihydrofolate reductase-thymidylate synthase gene of Plasmodium falciparum. Proc Natl Acad Sci U S A.

[5] Wilson CM, Serrano AE, Wasley A, Bogenschutz MP, Shankar AH, Wirth DF (1989). Amplification of a gene related to mammalian mdr genes in drug-resistant Plasmodium falciparum. Science.

[6] Guler JL, Freeman DL, Ahyong V, Patrapuvich R, White J, Gujjar R (2013). Asexual populations of the human malaria parasite, Plasmodium falciparum, use a two-step genomic strategy to acquire accurate, beneficial DNA amplifications. PLoS Pathog.

[7] Anderson TJ, Patel J, Ferdig MT (2009). Gene copy number and malaria biology. Trends Parasitol.

[8] Douglas RG, Amino R, Sinnis P, Frischknecht F (2015). Active migration and passive transport of malaria parasites. Trends Parasitol.

[9] Prudencio M, Rodriguez A, Mota MM (2006). The silent path to thousands of merozoites: the Plasmodium liver stage. Nat Rev Microbiol.

[10] Mutapi F, Billingsley PF, Secor WE (2013). Infection and treatment immunizations for successful parasite vaccines. Trends Parasitol.

[11] Nussenzweig RS, Vanderberg J, Most H, Orton C (1967). Protective immunity produced by the injection of x-irradiated sporozoites of plasmodium berghei. Nature.

[12] Hewitson JP, Hamblin PA, Mountford AP (2005). Immunity induced by the radiation-attenuated schistosome vaccine. Parasite Immunol.

[13] Silvie O, Semblat JP, Franetich JF, Hannoun L, Eling W, Mazier D (2002). Effects of irradiation on Plasmodium falciparum sporozoite hepatic development: implications for the design of pre-erythrocytic malaria vaccines. Parasite Immunol.

[14] Mueller AK, Labaied M, Kappe SH, Matuschewski K (2005). Genetically modified Plasmodium parasites as a protective experimental malaria vaccine. Nature.

[15] van Dijk MR, Douradinha B, Franke-Fayard B, Heussler V, van Dooren MW, van Schaijk B (2005). Genetically attenuated, P36p-deficient malarial sporozoites induce protective immunity and apoptosis of infected liver cells. Proc Natl Acad Sci U S A.

[16] Khan SM, Janse CJ, Kappe SH, Mikolajczak SA (2012). Genetic engineering of attenuated malaria parasites for vaccination. Curr Opin Biotechnol.

[17] Vaughan AM, O'Neill MT, Tarun AS, Camargo N, Phuong TM, Aly AS (2009). Type II fatty acid synthesis is essential only for malaria parasite late liver stage development. Cell Microbiol.

[18] Silvie O, Goetz K, Matuschewski K (2008). A sporozoite asparagine-rich protein controls initiation of Plasmodium liver stage development. PLoS Pathog.

[19] Nganou-Makamdop K, Sauerwein RW (2013). Liver or blood-stage arrest during malaria sporozoite immunization: the later the better?. Trends Parasitol.

[20] Matuschewski K, Hafalla JC, Borrmann S, Friesen J (2011). Arrested Plasmodium liver stages as experimental anti-malaria vaccines. Hum Vaccin.

[21] Spring M, Murphy J, Nielsen R, Dowler M, Bennett JW, Zarling S (2013). First-in-human evaluation of genetically attenuated Plasmodium falciparum sporozoites administered by bite of Anopheles mosquitoes to adult volunteers. Vaccine.

[22] Mikolajczak SA, Lakshmanan V, Fishbaugher M, Camargo N, Harupa A, Kaushansky A (2014). A next-generation genetically attenuated Plasmodium falciparum parasite created by triple gene deletion. Mol Ther.

[23] Lee AH, Symington LS, Fidock DA (2014). DNA repair mechanisms and their biological roles in the malaria parasite Plasmodium falciparum. Microbiol Mol Biol Rev.

[24] de Koning-Ward TF, Gilson PR, Crabb BS (2015). Advances in molecular genetic systems in malaria. Nat Rev Microbiol.

[25] Fox BA, Ristuccia JG, Gigley JP, Bzik DJ (2009). Efficient gene replacements in Toxoplasma gondii strains deficient for nonhomologous end joining. Eukaryot Cell.

[26] Gardner MJ, Hall N, Fung E, White O, Berriman M, Hyman RW (2002). Genome sequence of the human malaria parasite Plasmodium falciparum. Nature.

[27] Kirkman LA, Lawrence EA, Deitsch KW (2014). Malaria parasites utilize both homologous recombination and alternative end joining pathways to maintain genome integrity. Nucleic Acids Res.

[28] Straimer J, Lee MC, Lee AH, Zeitler B, Williams AE, Pearl JR (2012). Site-specific genome editing in Plasmodium falciparum using engineered zinc-finger nucleases. Nat Methods.

[29] Lasonder E, Janse CJ, van Gemert GJ, Mair GR, Vermunt AM, Douradinha BG (2008). Proteomic profiling of Plasmodium sporozoite maturation identifies new proteins essential for parasite development and infectivity. PLoS Pathog.

[30] Simonetti AB, Billingsley PF, Winger LA, Sinden RE (1993). Kinetics of expression of two major Plasmodium berghei antigens in the mosquito vector, Anopheles stephensi. J Eukaryot Microbiol.

[31] Tarun AS, Peng X, Dumpit RF, Ogata Y, Silva-Rivera H, Camargo N (2008). A combined transcriptome and proteome survey of malaria parasite liver stages. Proc Natl Acad Sci U S A.

[32] Matuschewski K, Ross J, Brown SM, Kaiser K, Nussenzweig V, Kappe SH (2002). Infectivity-associated changes in the transcriptional repertoire of the malaria parasite sporozoite stage. J Biol Chem.

[33] Silvie O, Briquet S, Muller K, Manzoni G, Matuschewski K (2014). Post-transcriptional silencing of UIS4 in Plasmodium berghei sporozoites is important for host switch. Mol Microbiol.

[34] Prado M, Eickel N, De Niz M, Heitmann A, Agop-Nersesian C, Wacker R (2015). Long-term live imaging reveals cytosolic immune responses of host hepatocytes against Plasmodium infection and parasite escape mechanisms. Autophagy.

[35] De Niz M, Helm S, Horstmann S, Annoura T, Del Portillo HA, Khan SM (2015). In vivo and in vitro characterization of a Plasmodium liver stage-specific promoter. PLoS One.

[36] Maeder ML, Thibodeau-Beganny S, Osiak A, Wright DA, Anthony RM, Eichtinger M (2008). Rapid "open-source" engineering of customized zinc-finger nucleases for highly efficient gene modification. Mol Cell.

[37] Deligianni E, Morgan RN, Bertuccini L, Kooij TW, Laforge A, Nahar C (2011). Critical role for a stage-specific actin in male exflagellation of the malaria parasite. Cell Microbiol.

[38] Carey AF, Singer M, Bargieri D, Thiberge S, Frischknecht F, Menard R (2014). Calcium dynamics of Plasmodium berghei sporozoite motility. Cell Microbiol.

[39] Donnelly ML, Hughes LE, Luke G, Mendoza H, ten Dam E, Gani D (2001). The 'cleavage' activities of foot-and-mouth disease virus 2A site-directed mutants and naturally occurring '2A-like' sequences. J Gen Virol.

[40] Miller JC, Holmes MC, Wang J, Guschin DY, Lee YL, Rupniewski I (2007). An improved zinc-finger nuclease architecture for highly specific genome editing. Nat Biotechnol.

[41] Sultan AA, Thathy V, Frevert U, Robson KJ, Crisanti A, Nussenzweig V (1997). TRAP is necessary for gliding motility and infectivity of plasmodium sporozoites. Cell.

[42] Braks JA, Franke-Fayard B, Kroeze H, Janse CJ, Waters AP (2006). Development and application of a positive–negative selectable marker system for use in reverse genetics in Plasmodium. Nucleic Acids Res.

[43] Nakamura Y, Gojobori T, Ikemura T (2000). Codon usage tabulated from international DNA sequence databases: status for the year 2000. Nucleic Acids Res.

[44] Puigbo P, Guzman E, Romeu A, Garcia-Vallve S. OPTIMIZER: a web server for optimizing the codon usage of DNA sequences. Nucleic Acids Res. 2007;35(Web Server issue):W126–31. doi:10.1093/nar/gkm219. 10.1093/nar/gkm219 PMC1933141 17439967

[45] Kumar KA, Baxter P, Tarun AS, Kappe SH, Nussenzweig V (2009). Conserved protective mechanisms in radiation and genetically attenuated uis3(−) and uis4(−) Plasmodium sporozoites. PLoS One.

[46] Tarun AS, Dumpit RF, Camargo N, Labaied M, Liu P, Takagi A (2007). Protracted sterile protection with Plasmodium yoelii pre-erythrocytic genetically attenuated parasite malaria vaccines is independent of significant liver-stage persistence and is mediated by CD8+ T cells. J Infect Dis.

[47] de Koning-Ward TF, Janse CJ, Waters AP (2000). The development of genetic tools for dissecting the biology of malaria parasites. Annu Rev Microbiol.

[48] Zhang M, Wang F, Li S, Wang Y, Bai Y, Xu X (2014). TALE: a tale of genome editing. Prog Biophys Mol Biol.

[49] McVey M, Lee SE (2008). MMEJ repair of double-strand breaks (director's cut): deleted sequences and alternative endings. Trends Genet.

[50] Mueller AK, Camargo N, Kaiser K, Andorfer C, Frevert U, Matuschewski K (2005). Plasmodium liver stage developmental arrest by depletion of a protein at the parasite-host interface. Proc Natl Acad Sci U S A.

[51] Sharma S, Javadekar SM, Pandey M, Srivastava M, Kumari R, Raghavan SC (2015). Homology and enzymatic requirements of microhomology-dependent alternative end joining. Cell Death Dis.

[52] Badugu SB, Nabi SA, Vaidyam P, Laskar S, Bhattacharyya S, Bhattacharyya MK (2015). Identification of Plasmodium falciparum DNA repair protein Mre11 with an evolutionarily conserved nuclease function. PLoS One.

[53] Paul K, Wang M, Mladenov E, Bencsik-Theilen A, Bednar T, Wu W (2013). DNA ligases I and III cooperate in alternative non-homologous end-joining in vertebrates. PLoS One.

[54] Wang M, Wu W, Wu W, Rosidi B, Zhang L, Wang H (2006). PARP-1 and Ku compete for repair of DNA double strand breaks by distinct NHEJ pathways. Nucleic Acids Res.

[55] Symington LS. End resection at double-strand breaks: mechanism and regulation. Cold Spring Harb Perspect Biol. 2014;6(8). doi:10.1101/cshperspect.a016436. 10.1101/cshperspect.a016436 PMC4107989 25085909

[56] Mateos-Gomez PA, Gong F, Nair N, Miller KM, Lazzerini-Denchi E, Sfeir A (2015). Mammalian polymerase theta promotes alternative NHEJ and suppresses recombination. Nature.

[57] Kent T, Chandramouly G, McDevitt SM, Ozdemir AY, Pomerantz RT (2015). Mechanism of microhomology-mediated end-joining promoted by human DNA polymerase theta. Nat Struct Mol Biol.

[58] Zhang C, Xiao B, Jiang Y, Zhao Y, Li Z, Gao H, et al. Efficient editing of malaria parasite genome using the CRISPR/Cas9 system. mBio. 2014;5(4):e01414-14. doi:10.1128/mBio.01414-14. 10.1128/mBio.01414-14 PMC4161241 24987097

[59] Peng D, Kurup SP, Yao PY, Minning TA, Tarleton RL. CRISPR-Cas9-mediated single-gene and gene family disruption in Trypanosoma cruzi. mBio. 2015;6(1):e02097-14. doi:10.1128/mBio.02097-14. 10.1128/mBio.02097-14 PMC4281920 25550322

[60] Religa AA, Ramesar J, Janse CJ, Scherf A, Waters AP (2014). P. berghei telomerase subunit TERT is essential for parasite survival. PLoS One.

[61] van Schaijk BC, Ploemen IH, Annoura T, Vos MW, Foquet L, van Gemert GJ, et al. A genetically attenuated malaria vaccine candidate based on P. falciparum b9/slarp gene-deficient sporozoites. eLife. 2014;3k. doi:10.7554/eLife.03582. 10.7554/eLife.03582 PMC4273440 25407681

[62] Janse CJ, Ramesar J, Waters AP (2006). High-efficiency transfection and drug selection of genetically transformed blood stages of the rodent malaria parasite Plasmodium berghei. Nat Protoc.

[63] Frischknecht F, Baldacci P, Martin B, Zimmer C, Thiberge S, Olivo-Marin JC (2004). Imaging movement of malaria parasites during transmission by Anopheles mosquitoes. Cell Microbiol.

[64] Schindelin J, Arganda-Carreras I, Frise E, Kaynig V, Longair M, Pietzsch T (2012). Fiji: an open-source platform for biological-image analysis. Nat Methods.

[65] Aurrecoechea C, Brestelli J, Brunk BP, Dommer J, Fischer S, Gajria B (2009). PlasmoDB: a functional genomic database for malaria parasites. Nucleic Acids Res.

