# Supplementary figures and images for: Zinc finger nuclease-based double-strand breaks attenuate malaria parasites and reveal rare microhomology-mediated end joining
Source: Genome Biol. 2015 Nov 17;16:249. doi: 10.1186/s13059-015-0811-1 (PMC4647826; doi:10.1186/s13059-015-0811-1)

A

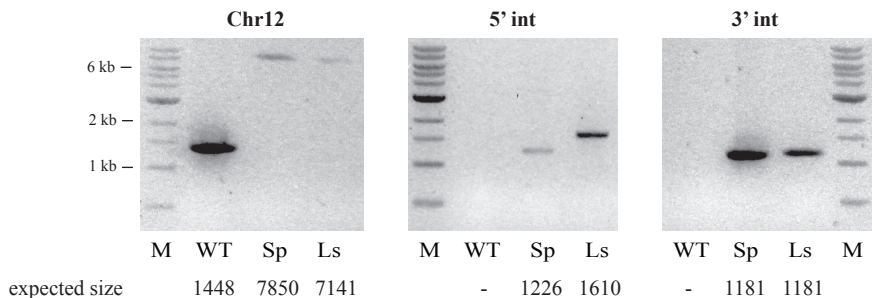

B

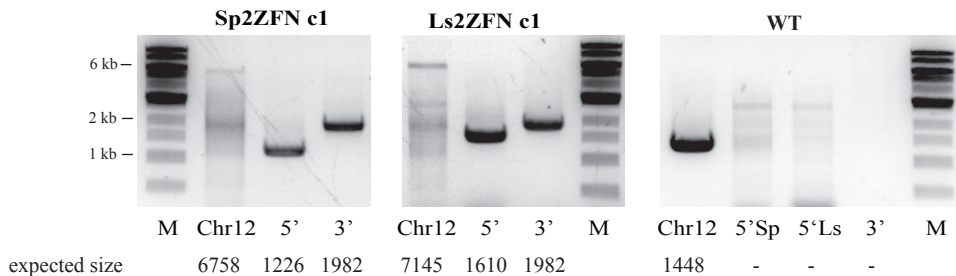

C

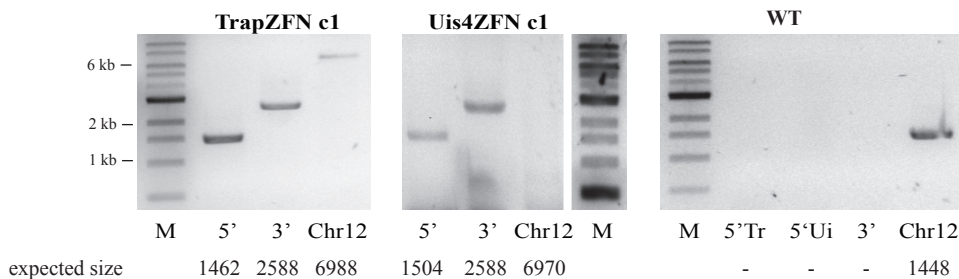

Supplement: Additional file 1: Fig. S1. — Integration PCRs of parasites used in this study. a Integration of SpZFN and LsZFN. PCR with P53/P54 amplifies the whole Chr12 locus, resulting in 1448 bp in WT, 7850 bp for SpZFN and 7141 bp for LsZFN. The 5’ integration was amplified with P53/P12 or P53/P24, resulting in 1226 bp and 1610 bp for SpZFN and LsZFN, respectively. The 3’ integration was performed with P54/P55, resulting in 1181 bp. b Integration of Sp2ZFN and Ls2ZFN. PCR of the whole locus resulted in 6758 bp or 7145 bp for Sp2ZFN and Ls2ZFN, respectively. The 5’ integration was performed with P53/P56 or P53/P24, resulting in 1226 bp or 1610 bp for Sp2ZFN and Ls2ZFN. The 3’ integration was verified with P54/P10 (1982 bp). c Integration of TrapZFN and Uis4ZFN. PCR of the whole locus resulted in 6988 bp and 6970 bp for TrapZFN and Uis4ZFN, respectively. The 5’ integration was performed with P53/P22 or P53/P38, resulting in 1462 bp and 1504 bp for TrapZFN and Uis4ZFN, respectively. The 3’ integration was verified with P54/P8 (2588 bp). (PDF 1088 kb) [file 13059_2015_811_MOESM1_ESM.pdf]

A

WT

CSP

Hoechst

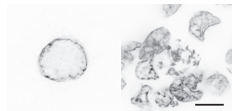

SpZFN SI 2

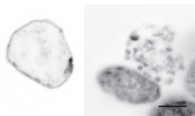

B

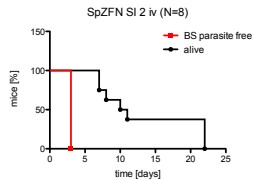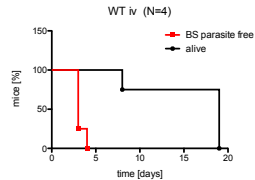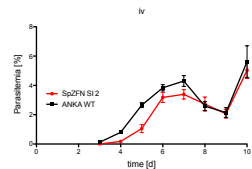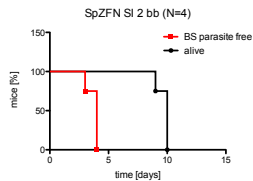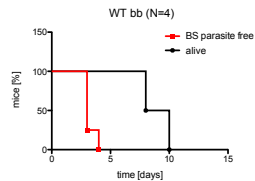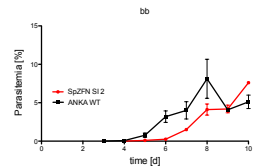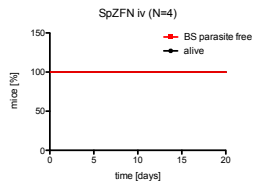

Supplement: Additional file 2: Fig. S2. — Characterisation of SpZFN SI 2. a Liver stages of parasites in HepG2 cells 48 h after sporozoite invasion. CSP staining shows the plasma membrane of the parasite within the hepatocyte. b C57BL/6 mice were challenged with 10,000 sporozoites i.v. or by the bites of ten infected A. stephensi mosquitoes (bb). Peripheral blood parasitaemia was monitored by Giemsa-stained blood smears from three days post-infection. Percentage of mice that are blood stage (BS) parasite free and percentage of mice alive is shown over time. Parasitaemia over the course of the experiments is shown as growth curves. Control infections with SpZFN resulted in no blood stage parasitaemia. (PDF 1601 kb) [file 13059_2015_811_MOESM2_ESM.pdf]

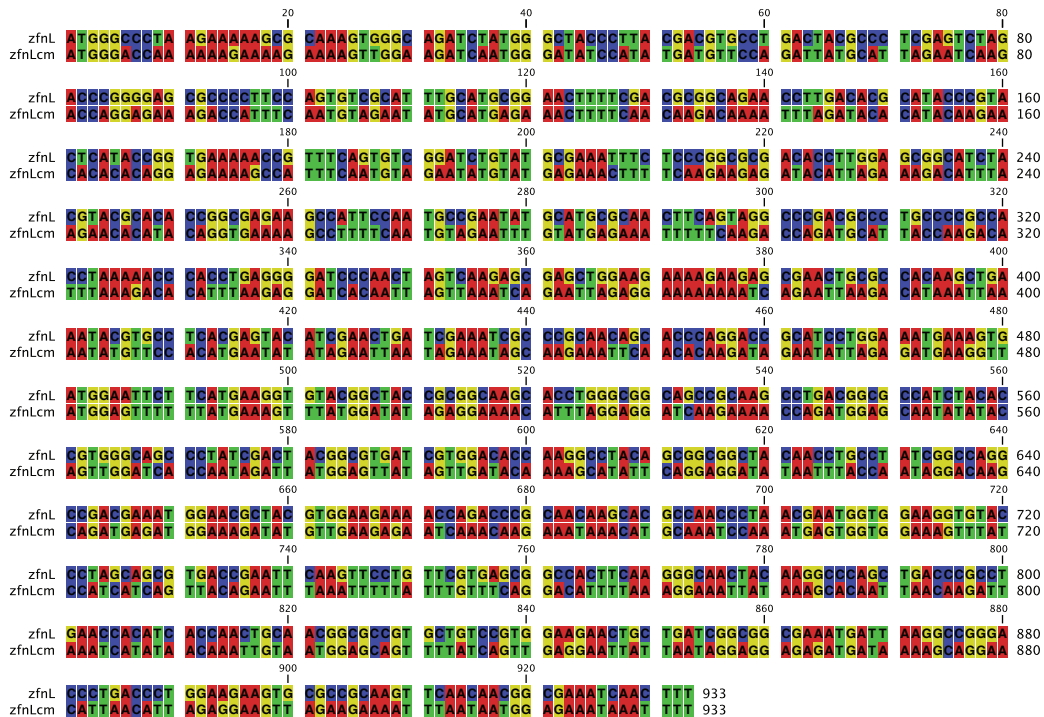

Supplement: Additional file 3: Fig. S3. — Alignment of zfnL and zfnLcm. (PDF 1188 kb) [file 13059_2015_811_MOESM3_ESM.pdf]

CSP/Uis4

GFP

Hoechst

Sp2ZFN

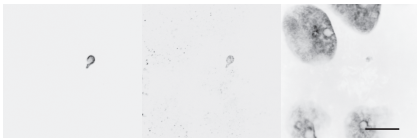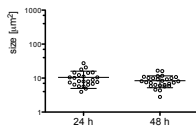

TRAPZFN

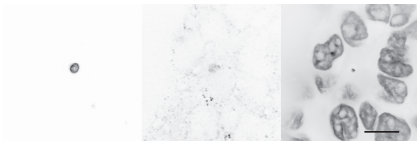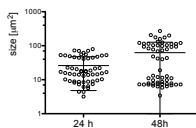

Uis4ZFN

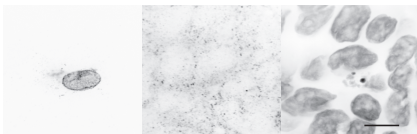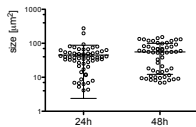

Ls2ZFN

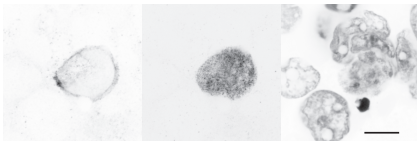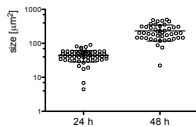

Supplement: Additional file 4: Fig. S4. — Liver stages of parasites in HepG2 cells 48 h after sporozoite invasion. CSP or Uis4 staining shows the plasma membrane of the parasite within the hepatocyte. Staining with α-GFP antibody shows residual expression of mGFP-hDHFR fusion protein. Sizes of liver stages from single slice images were counted 24 h and 48 h post-sporozoite invasion. (PDF 7464 kb) [file 13059_2015_811_MOESM4_ESM.pdf]

A

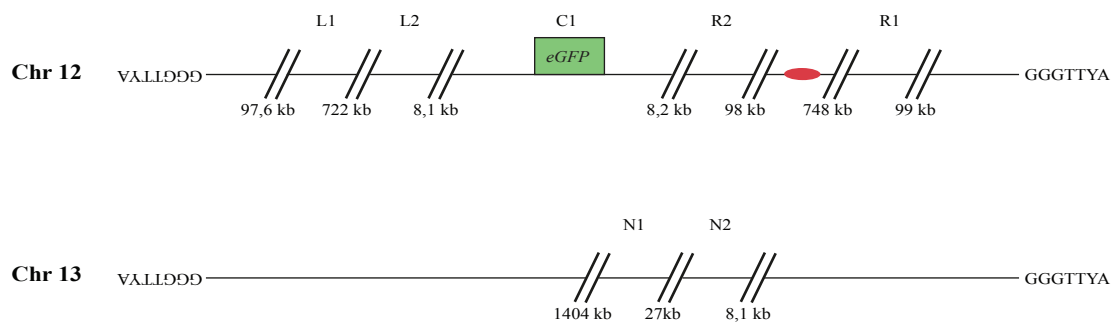

B

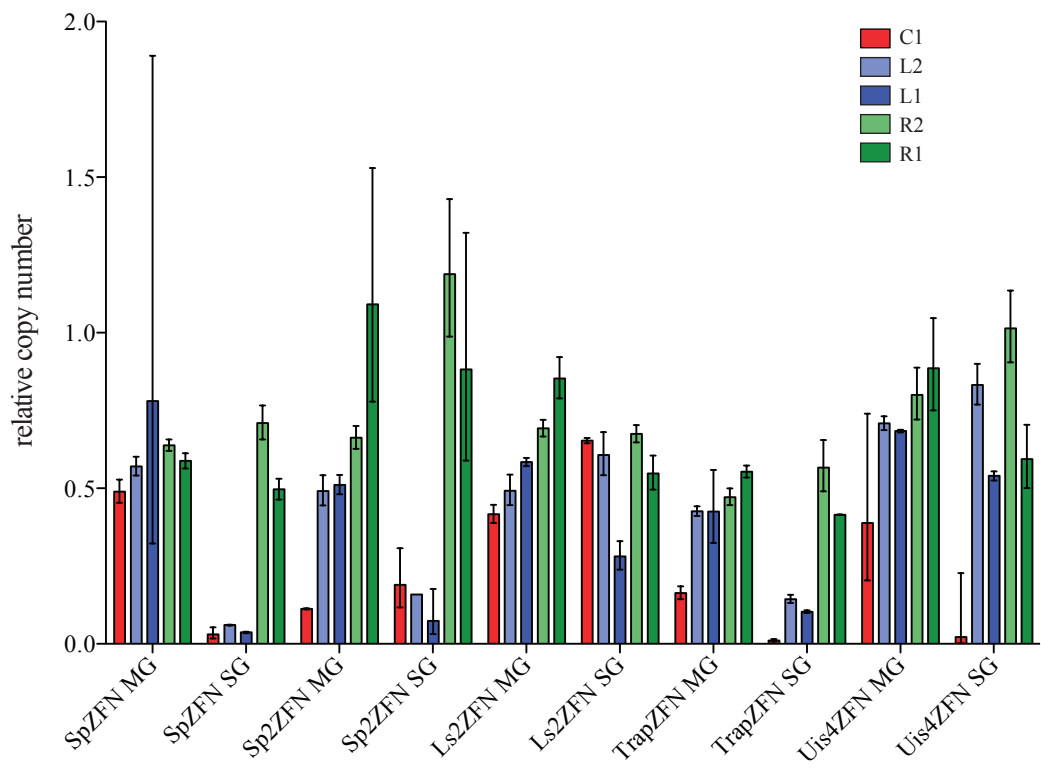

Supplement: Additional file 6: Fig. S6. — Results of qPCR on gDNA. a Localisation of qPCR probes on chromosomes 12 and 13. The centromere of chromosome 12 is shown in red. Binding sites for primer pairs used in qPCR are shown. Primer pair C1 amplifies the product over the cutting site of the ZFNs, while primer pairs L1 and R1 bind approximately 100 kb away from the telomeres on the left and right arm of chromosome 12, respectively. L2 and R2 bind around 8 kb away from the cutting site. N1 and N2 bind on the ‘control’ chromosome 13 and are used for normalization. b Chromosome 12 integrity is shown for samples from salivary gland (SG) and midgut (MG). Values were normalised to N1 and N2 on chromosome 13 and to results from blood-stage gDNA amplification. Positive and negative error is calculated from standard error of the mean from technical duplicates. (PDF 350 kb) [file 13059_2015_811_MOESM6_ESM.pdf]
