# Supplementary material for: Zinc finger nuclease-based double-strand breaks attenuate malaria parasites and reveal rare microhomology-mediated end joining
Source: Genome Biol. 2015 Nov 17;16:249. doi: 10.1186/s13059-015-0811-1 (PMC4647826; doi:10.1186/s13059-015-0811-1)
Supplement: Additional file 5: Fig. S5. — Overview of all microhomology sequences used by SI parasites to repair DSBs. Microhomology sequences are shown in bold. Ten base pairs flanking each homology region are shown prior to the DSB break and the remaining bases in the SI parasites. The number of base pairs lost after repair is indicated. The mismatch in the microhomology sequence used for repair of Uis4ZFN SI 1–3 is highlighted in orange. (PDF 280 kb) [file 13059_2015_811_MOESM5_ESM.pdf]

SpZFN	CTATATCATG	**GCCGAC**	AAGCAGAAGA	–/ /–	CGTGCAGCTC	**GCCGAC**	CACTACCAGC
SpZFN SI 1–4	CTATATCATG	**GCC**		–/75 bp/–		**GAC**	CACTACCAGC
SpZFN	ATGGCCGACA	**AGCAGAA**	GAACGGCATC	–/ /–	GACCACTACC	**AGCAGAA**	CACCCCCATC
SpZFN SI 8c1	ATGGCCGACA	**AGCA**		–/81 bp/–		**GAA**	CACCCCCATC
LsZFN	AGAAGAACGG	**CATC**	AAGGTGAACT	–/ /–	AGAACACCCC	**CATC**	GGCGACGGCC
LsZFN SI 3	AGAAGAACGG	**CA**		–/81 bp/–		**TC**	GGCGACGGCC
TrapZFN	TCTGCACCAC	**CGGCA**	AGCTGCCCGT	–/ /–	ACATCGAGGA	**CGGCA**	GCGTGCAGCT
TrapZFN SI 1	TCTGCACCAC	**CGG**		–/369 bp/–		**GCA**	GCGTGCAGCT
TrapZFN	TCAGGGTGTC	**CGGCGA**	GGGCGAGGGC	–/ /–	ACACCCCCAT	**CGGCGA**	CGGCCCCGTG
TrapZFN SI 2	TCAGGGTGTC	**CGG**		–/474 bp/–		**CGA**	CGGCCCCGTG
Uis4ZFN	ATCATGGCAG	**ACAAGCAGAA**	GAACGGCATC	–/ /–	GCCGACCACT	**ACCAGCAGAA**	CACCCCCATC
Uis4ZFN SI 1–2	ATCATGGCAG			–/81 bp/–		**ACCAGCAGAA**	CACCCCCATC
Uis4ZFN SI 3	ATCATGGCAG	**ACA**		–/81 bp/–		**AGCAGAA**	CACCCCCATC
