# Supplementary material for: Zinc finger nuclease-based double-strand breaks attenuate malaria parasites and reveal rare microhomology-mediated end joining
Source: Genome Biol. 2015 Nov 17;16:249. doi: 10.1186/s13059-015-0811-1 (PMC4647826; doi:10.1186/s13059-015-0811-1)
Supplement: Additional file 7: Fig. S7. — Map of chromosomal location of multiplex target sites using CAS9. a All perfect matches for the possible gRNA sequence TTATATTAGTATTCGTTATTTGG targeting 104 loci of the P. berghei genome in different bir genes and bir pseudogenes. Archived contigs represent sequences that have not successfully been mapped to a chromosome. b The same sequence targets 532 targets in the P. yoelii genome within 522 different yir and yir pseudogenes. c Possible gRNA sequence GGTAACAACACAACAGCTAGTGG matches 39 targets within 31 var genes in P. falciparum. Figures were created with Plasmodb.org [65]. (PDF 996 kb) [file 13059_2015_811_MOESM7_ESM.pdf]

A

Sequence	Organism	Chromosome	#Genomic Segments	Length	Genomic Segment Locations
PbANKA_11_v3	*Plasmodium berghei ANKA*	11	14	1762642	
PbANKA_00_v3_archived_contig_2	*Plasmodium berghei ANKA*		11	34265	
PbANKA_07_v3	*Plasmodium berghei ANKA*	07	10	846620	
PbANKA_14_v3	*Plasmodium berghei ANKA*	14	10	2549703	
PbANKA_08_v3	*Plasmodium berghei ANKA*	08	9	1420537	
PbANKA_12_v3	*Plasmodium berghei ANKA*	12	9	1807262	
PbANKA_00_v3_archived_contig_1	*Plasmodium berghei ANKA*		6	56810	
PbANKA_06_v3	*Plasmodium berghei ANKA*	06	6	984266	
PbANKA_09_v3	*Plasmodium berghei ANKA*	09	6	1633586	
PbANKA_03_v3	*Plasmodium berghei ANKA*	03	5	659512	
PbANKA_10_v3	*Plasmodium berghei ANKA*	10	4	1640193	
PbANKA_13_v3	*Plasmodium berghei ANKA*	13	4	2521873	
PbANKA_00_v3_archived_contig_5	*Plasmodium berghei ANKA*		3	6544	
PbANKA_00_v3_archived_contig_3	*Plasmodium berghei ANKA*		2	24479	
PbANKA_02_v3	*Plasmodium berghei ANKA*	02	2	622508	
PbANKA_05_v3	*Plasmodium berghei ANKA*	05	2	931174	
PbANKA_01_v3	*Plasmodium berghei ANKA*	01	1	515659	
Sequence	Organism	Chromosome	#Genomic Segments	Length	Genomic Segment Locations

B

Sequence	Organism	Chromosome	#Genes	Length	Gene Locations
Py17X_13_v2	*Plasmodium yoelii yoelii 17X*	13	27	2795668	
Py17X_12_v2	*Plasmodium yoelii yoelii 17X*	12	24	1978248	
Py17X_01_v2	*Plasmodium yoelii yoelii 17X*	01	23	703413	
Py17X_04_v2	*Plasmodium yoelii yoelii 17X*	04	22	861284	
Py17X_09_v2	*Plasmodium yoelii yoelii 17X*	09	22	1824586	
Py17X_bin_v2_62_716070-865457	*Plasmodium yoelii yoelii 17X*		22	149388	
Py17X_10_v2	*Plasmodium yoelii yoelii 17X*	10	21	1757347	
Py17X_11_v2	*Plasmodium yoelii yoelii 17X*	11	19	1857262	
Py17X_bin_v2_52_282045-383119	*Plasmodium yoelii yoelii 17X*		16	101075	
Py17X_06_v2	*Plasmodium yoelii yoelii 17X*	06	15	1061054	
Py17X_14_v2	*Plasmodium yoelii yoelii 17X*	14	15	2590618	
Py17X_08_v2	*Plasmodium yoelii yoelii 17X*	08	14	1528798	
Py17X_02_v2	*Plasmodium yoelii yoelii 17X*	02	12	710674	
Py17X_03_v2	*Plasmodium yoelii yoelii 17X*	03	12	706722	
Py17X_05_v2	*Plasmodium yoelii yoelii 17X*	05	12	1051162	
Py17X_bin_v2_58_553689-620495	*Plasmodium yoelii yoelii 17X*		12	66807	
Py17X_bin_v2_56_472671-540670	*Plasmodium yoelii yoelii 17X*		11	68000	
Py17X_bin_v2_65_895141-930446	*Plasmodium yoelii yoelii 17X*		10	35306	
Py17X_bin_v2_75_1171049-1260787	*Plasmodium yoelii yoelii 17X*		10	89739	
Py17X_bin_v2_80_1413295-1479480	*Plasmodium yoelii yoelii 17X*		10	66186	
Py17X_07_v2	*Plasmodium yoelii yoelii 17X*	07	8	891477	
Py17X_bin_v2_51_229399-282044	*Plasmodium yoelii yoelii 17X*		8	52646	
Py17X_bin_v2_68_965039-1011924	*Plasmodium yoelii yoelii 17X*		8	46886	
Py17X_bin_v2_79_1361008-1413294	*Plasmodium yoelii yoelii 17X*		8	52287	
Py17X_bin_v2_59_620496-659553	*Plasmodium yoelii yoelii 17X*		7	39058	
Sequence	Organism	Chromosome	#Genes	Length	Gene Locations

C

Sequence	Organism	Chromosome	#Genomic Segments	Length	Genomic Segment Locations
Pf3D7_07_v3	*Plasmodium falciparum 3D7*	7	7	1445207	
Pf3D7_08_v3	*Plasmodium falciparum 3D7*	8	6	1472805	
Pf3D7_04_v3	*Plasmodium falciparum 3D7*	4	5	1200490	
Pf3D7_12_v3	*Plasmodium falciparum 3D7*	12	5	2271494	
Pf3D7_01_v3	*Plasmodium falciparum 3D7*	1	3	640851	
Pf3D7_03_v3	*Plasmodium falciparum 3D7*	3	3	1067971	
Pf3D7_06_v3	*Plasmodium falciparum 3D7*	6	3	1418242	
Pf3D7_10_v3	*Plasmodium falciparum 3D7*	10	2	1687656	
Pf3D7_13_v3	*Plasmodium falciparum 3D7*	13	2	2925236	
Pf3D7_02_v3	*Plasmodium falciparum 3D7*	2	1	947102	
Pf3D7_05_v3	*Plasmodium falciparum 3D7*	5	1	1343557	
Pf3D7_09_v3	*Plasmodium falciparum 3D7*	9	1	1541735	
Sequence	Organism	Chromosome	#Genomic Segments	Length	Genomic Segment Locations
