# Supplementary material for: Zinc finger nuclease-based double-strand breaks attenuate malaria parasites and reveal rare microhomology-mediated end joining
Source: Genome Biol. 2015 Nov 17;16:249. doi: 10.1186/s13059-015-0811-1 (PMC4647826; doi:10.1186/s13059-015-0811-1)
Supplement: Additional file 8: Fig. S8. — A list of all primers used in this study. (PDF 396 kb) [file 13059_2015_811_MOESM8_ESM.pdf]

Primers used in this study

P1 Sp	ATAAGAATGCGGCCGCATGTGTTGGTTGTAATTGAGG
P1 Ls	AAGGAAAAAAGCGGCCGCGTTGCATTATCGTCAAAAGTG
P1 Uis4	AAGGAAAAAAGCGGCCGCGCTCATTATAGGGTGAATAAAAATGG
P1 Trap	AAGGAAAAAAGCGGCCGCGCTCATATTAATACACATAATGTCTTCC
P2	CGGGATATCATATTTGTAATGATGCTTTTTCACG
P 3	CTTGCACCGGTATGGTGAGCAAGGGCGAG
P4	CTTGCACCGGTACCACCACCACCACCACCACCACCACCACCTCTAGATCCGGTGGATCCC
P5	GGAATTCCCAATTCTTTTCGAGCTCTTTATGC
P6	GGAATTCCATATGCGCTTTTATAAAATTTTTATTTATTTATAAGC
P7	CTTGCACCGGTATGGTTGGTTCGCTAAAC
P8	CTAGCTAGCTTAATCATTCTTCTCATATACTTC
P9	CTTGCACCGGTATGGTGAGCAAGGGCGAG
P10	CTTGCACCGGTACCACCACCACCACCACCACCACCACCACCTCTAGATCCGGTGGATCCC
P11	ATAAGAATGCGGCCGCATGTGTTGGTTGTAATTGAGG
P12	CCGGATATCCGTATTTAAATACGGACAATTGTCGGGGTACCTTTAAATATATGCGTGTATATATAGATTTTG
P13	CGGGGTACCATGGGCCCTAAGAAAAAGC
P14	ATACGGACAATTGTCTTAAAAGTTGATTTCGCCGTTG
P15	ATACGGACAATTGTCGTATTCTTTAATTAAATAAACATTACGCATG
P16	ACGTATTTAAATGTCATAGCAAGTTAACTACATATATAATAAAC
P17	ACGTATTTAAATACGGACAATTGTCGGGGTACCTAGCTAAAAGGTGTGCAAGC
P18	CGGGATATCATATTTGTAATGATGCTTTTTCACG
P19	GACAATTGTCATGGGCCCTAAGAAAAAGC
P20	CGGGGTACCTTAAAAGTTGATTTCGCCGTTG
P21	ACGTATTTAAATGCTCATATTAATACACATAATGTCTTCC
P22	GACAATTGTCTATAAGGGAAAGGGAAAATGGG
P23	AAGGAAAAAAGCGGCCGCGTTGCATTATCGTCAAAAGTG
P24	GCTTTTTCTTAGGGCCCATTTTTTATGTGTAAAAAAGTAAAATGATTATAATAGAAG
P25	CTTCTATTATAATCATTTTACTTTTTTACACATAAAAAATGGGCCCTAAGAAAAAGC
P26	ATACGGACAATTGTCCCTGGACCTGGATTTCTTCAACATCACCACATGTAAGAAGTGAACCTCTACCTTCACCAAAGTTGATTTCGCCGTTG
P27	AATCTGCAGAATTCGAAGCTTGAGC
P28	GTCTATATCATGGCAGACAAGCAGAAGAACGGC
P29	CTTCTGCTTGTCTGCCATGATATAGACGTTGTG
P30	TTAGATTTAAATTTATTTTAATATTTCCTAAAATATATATACTTTGTG
P31	GCGGCCGCATGTGTTGGTTGTAATTGAGG
P32	ATTAAGCTTTTTAAATATATGCGTGTATATATAGATTTTG
P33	GCGGCCGCGTTGCATTATC
P34	ATTAAGCTTTTTATGTGTAAAAAAGTAAAATGATTATAATAGAAG
P35	AAGGAAAAAAGCGGCCGCGCTCATATTAATACACATAATGTCTTCC
P36	GGGAATTCCATATGGATATCCCATTGATCTTCCAACTTTTCTTTTCTTTTTTGGTCCCATTCTATAAGGGAAAGGGAAAATGG
P37	AAGGAAAAAAGCGGCCGCGCTCATTATAGGGTGAATAAAAATGG
P38	GGGAATTCCATATGGATATCCCATTGATCTTCCAACTTTTCTTTTCTTTTTTGGTCCCATTTATTCAGACGTAATAATTATGTGC
P39	TAGTGGTTGTCGGGCAGCAG
P40	ACAACTACAACAGCCACAACGTC
P41	TAGTGGTTGTCGGGCAGCAG
P42	GCAATTTGGTTCACTTTCGCCAATG
P43	GGTACACCTCCTCAAGAAATTCATCC
P44	ACCAGTTATTGACCCAGATCCAAAAAC
P45	GCGTTATCTTCTCGGCGTAATACC
P46	AATTTGGAATTGTTCAAATGCTGGATTG
P47	TGCTTAATCAATATTTTTCTTCTATGACCAGAAC
P48	AAAATTATGAAAGACCCTACTGTAAACACATC
P49	TGTACGAAGGAAATCCAAATTCTTCTACTC
